# UGRP1-modulated MARCO^+^ alveolar macrophages contribute to age-related lung fibrosis

**DOI:** 10.1186/s12979-023-00338-8

**Published:** 2023-03-18

**Authors:** Yongyan Chen, Xiaolei Hao, Ming Li, Zhigang Tian, Min Cheng

**Affiliations:** 1 Hefei National Laboratory for Physical Sciences at Microscale, the CAS Key Laboratory of Innate Immunity and Chronic Disease, School of Basic Medical Sciences, Division of Life Sciences and Medicine, University of Science and Technology of China, Hefei, 230027 China; 2 Institute of Immunology, University of Science and Technology of China, Hefei, 230027 China; 3 Department of Pathology, The Second Affiliated Hospital of Anhui Medical University, Hefei, 230601 Anhui China; 4 Cancer Immunotherapy Center, the First Affiliated Hospital of University of Science and Technology of China (Anhui Provincial Hospital), Hefei, 230001 China; 5 Department of Geriatrics, Gerontology Institute of Anhui Province, The First Affiliated Hospital of USTC, Division of Life Sciences and Medicine, University of Science and Technology of China, Hefei, 230001 China; 6 Anhui Provincial Key Laboratory of Tumor Immunotherapy and Nutrition Therapy, Hefei, 230001 China

**Keywords:** Age-related lung fibrosis, Alveolar macrophage, UGRP1, MARCO, CCL6

## Abstract

The aging lungs are vulnerable to chronic pulmonary diseases; however, the underlying mechanisms are not well understood. In this study, we compared the aging lungs of 20–24-month-old mice with the young of 10–16-week-old mice, and found that aging airway epithelial cells significantly upregulated the expression of uteroglobin-related protein 1 (UGRP1), which was responsible for the higher levels of CCL6 in the aging lungs. Alveolar macrophages (AMs) changed intrinsically with aging, exhibiting a decrease in cell number and altered gene expression. Using terminal differentiation trajectories, a population of MARCO^+^ AMs with the ability to produce CCL6 was identified in the aging lungs. Upregulated UGRP1was demonstrated to modulate CCL6 production of AMs in the UGRP1-MARCO pair in vivo and in vitro. Furthermore, MARCO^+^ AMs aggravated bleomycin-induced pulmonary fibrosis in a CCL6-dependent manner in the aged mice, and blocking MARCO or neutralizing CCL6 significantly inhibited pulmonary fibrosis, similar to the depletion of AMs. The age-related upregulation of UGRP1 and MARCO^+^ AMs, involved in the progression of lung fibrosis, was also observed in human lung tissues. Thus, UGRP1 modulated MARCO^+^ AMs regarding the age-related lung fibrosis in a CCL6-dependent manner, which is key to establishing optimal targeting for the aging population.

**Supplementary Information:**

The online version contains supplementary material available at 10.1186/s12979-023-00338-8.

## Introduction

The lung is an organ susceptible to natural aging, which is associated with declined lung function, diminished pulmonary remodeling and regeneration capacity, and enhanced susceptibility to pulmonary diseases such as chronic obstructive pulmonary disease (COPD), pulmonary fibrosis, cancer and infections [1, 2]. Chronic lower respiratory disease is reported as the third leading cause of death in people (≥ 65 years) worldwide [3]. With the rapid increase in the aging population, it is crucial to explore what alterations in cellular function and cross-talk of pulmonary resident cells and immune cells contribute to the development and progression of pulmonary diseases in the aging lungs [1].

Alveolar macrophages (AMs) are the most abundant innate immune cells, accounting for approximately 90% of resident immune cells in the lungs, located on the luminal surface of the alveolar space [1, 4]. Notably, the state and function of AMs are shaped by the ontogeny and local environment. As the lung’s first “wound”, the first breath of a newborn creates the formation of alveolar niche, which gets rapidly populated by circulating fetal monocytes that differentiate into AMs and subsequently engrafts themselves into the alveolar niche, dependent on granulocyte–macrophage colony-stimulating factor (GM-CSF) produced by alveolar type II cells [4]. AMs are long-lived, with a turnover rate of only ~ 40% in one year; however, their phenotype and function are considerably influenced by the microenvironment, such as common microbiota, pathogen infections, and lung inflammation and injury [5–8].

The early unresponsiveness of neonatal AMs was demonstrated to be both intrinsic and related to the immunosuppressive environment in neonatal lungs, which can be regulated by microbial exposure early in life [9, 10]. In adults, AMs are characterized as F4/80^+^ CD11c^+^ with high expression levels of CD200R, CD206, and Siglec-F and low expression levels of major histocompatibility complex (MHC)-II and co-stimulation molecules, which are distinguished from the other macrophage populations [5]. With aging, the phagocytosis of apoptotic neutrophils by AMs becomes defective due to the downregulated expression of CD204, which induces the retention of neutrophils and contributes to more severe lung damage during influenza infections in elderly individuals [11]. Moreover, AM phagocytosis of bacteria is impaired by aging through the reduction in the cell surface expression of the macrophage receptor with collagenous structure (MARCO), a bacterial scavenger receptor, that interacts with bacteria to initiate cytoskeleton remodeling and phagocytosis in AMs [12]. In the AMs of aged mice, suppressed Rac1-GTP signaling was demonstrated to decrease actin-related protein-2/3 activation, and subsequently attenuate F-actin polymerization, filopodia formation and MARCO expression [12]. Changes in AMs with advancing age, including the age-related resistance of AMs to proliferation and to GM-CSF signaling, were demonstrated not to be cell autonomous, but instead to be determined by their resident alveolar microenvironment, independent of circulating signaling molecules or cells [13]. However, it is not clear how alterations occur in the interactions of pulmonary resident cells and AMs in the aging lungs.

In the steady state, AMs are in close contact with the respiratory epithelium, with interactions occurring through CD200R, transforming growth factor (TGF)-β and interleukin (IL)-10R [5]. Notably, a lung-specific ligand-receptor pair demonstrated that uteroglobin-related protein 1 (UGRP1), only expressed in bronchial epithelial Clara-like cells in the lung tissue, is the ligand specific to the receptor MARCO expressed by AMs [14]. The increased expression of UGRP1 in cystic fibrosis, asthma and rhinitis suggested that UGRP1-MARCO be involved in these inflammatory diseases [14, 15]. In a mouse model of airway allergy, UGRP1 can suppress inflammation by markedly reducing the infiltration of eosinophils in lung tissue, and the levels of proinflammatory cytokines IL-4, IL-5 and IL-13 in bronchoalveolar lavage fluids (BALF) [16]. Moreover, the expression of MARCO in AMs was regulated by factors in the lung tissue microenvironment, such as tumor cell-derived IL-37 or bacterial induced IL-10 [17, 18]. In the aged lung, respiratory airway epithelial cells change with regard to their surfactant composition, exhibiting increased oxidative stress, decreased cell renewal, increased apoptosis, and enhanced senescence [1, 2]. Thus, the expression of UGRP1 and its interaction with MARCO in the aging lung deserve further investigation.

In this study, a population of MARCO^+^ AMs with the ability to produce CCL6 was identified in aged mice. Furthermore, UGRP1 upregulated by aging epithelial cells modulated the function of AMs in UGRP1-MARCO pair, which accounted for the enhanced susceptibility to pulmonary fibrosis in aged individuals.

## Results

### Aging airway epithelial cells upregulate UGRP1 expression responsible for the high level of CCL6 in the lungs

**Fig. 1:**
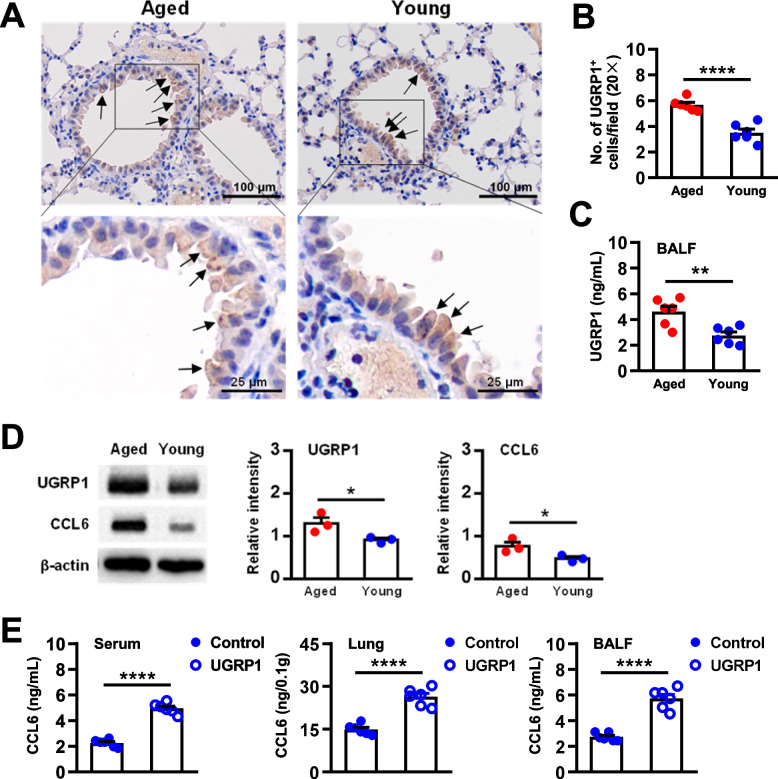
Airway epithelial cells upregulated UGRP1 expression responsible for the high level of CCL6 in the aged lungs. The lung tissues of aged mice (20–24 months old) were compared with those of young mice (10–16 weeks old). **A** Expression levels of UGRP1 in airway epithelial cells detected by immunohistochemistry. The arrows indicate the UGRP1 positive cells in the lung tissues. Scale bar, 100 μm (upper) or 25 μm (blow). **B** UGRP1^+^ cell numbers in the lung tissues. Each symbol represents the average of 10 fields of vision (20 ×) from an individual sample. **C** The expression levels of UGRP1 in the bronchoalveolar lavage fluid (BALF, 1.0 mL/mouse) were detected by ELISA. There were 6 mice in each group. **D** Expression levels of UGRP1 and CCL6 in the lung tissues detected by western blotting. Data were normalized relative to the expression level of β-actin protein in the same sample. There were 3 mice in each group. **E** Young mice were treated with UGRP1 (15 μg/mouse, once a week for two weeks), and then the expression levels of CCL6 in the serum, lung tissues and BALF (1.0 mL/mouse) were detected by ELISA. There were 6 mice in each group. Data are shown as the mean ± SEM. Comparisons by unpaired two-tailed Student’s *t*-test. *p < 0.05, **p < 0.01, **** *p* < 0.0001

As previously reported [14], airway epithelial cells expressed UGRP1 (Fig. 1A). Compared with the young lung tissue, a larger number of UGRP1^+^ epithelial cells were observed in the aged lung tissue by immunohistochemistry (IHC) analysis (Fig. 1A and B); and higher protein levels of UGRP1 were detected in lung bronchoalveolar lavage fluid (BALF) of the aged mice (Fig. 1C). The protein expression level of UGRP1 was significantly increased in the aged lung tissue, and interestingly the protein expression level of C–C motif chemokine ligand 6 (CCL6) was consistently increased in the aged lung tissue (Fig. 1D). When UGRP1 was administrated to the young mice, the expression levels of CCL6 in the serum, lung tissue and BALF were significantly increased, demonstrating the critical role of UGRP1 in the induction of CCL6 in the lungs (Fig. 1E). Senescent airway epithelial cells owned indicated alterations, which would affect the regional immune environment of lung tissue.

### AMs are intrinsically altered by aging with regard to cell number and gene expression

The frequency and number of lung-resident AMs were significantly decreased in the aged mice compared with young mice, and the decrease was not affected in the co-housed aged mice with the young mice, indicating that AMs were intrinsically altered by aging (Supplemental Fig. 1A). Subsequently, AMs were purified (Supplemental Fig. 1B); and then analyzed by mRNA sequencing. There were 1298 differentially expressed genes (DEGs) between the aged and young AMs, with 996 upregulated genes (76.73%) and 302 downregulated genes (23.27%) (Supplemental Fig. 1C and D). Gene Ontology (GO) enrichment analysis showed that these DEGs were mainly enriched in 30 biological pathways including cell junction/adhesion/migration/motility, cell proliferation/activation/differentiation, cytokine secretion, and chemotaxis (Supplemental Fig. 1E). The Kyoto Encyclopedia of Genes and Genomes (KEGG) enrichment analysis likewise demonstrated that the DEGs were predominantly enriched in 20 pathways, including cell adhesion molecules, focal adhesion, cytokine-cytokine receptor interaction, biosynthesis, and metabolism (Supplemental Fig. 1F). Furthermore, AMs of the aged mice exhibited both M1 and M2 features, as shown by the enhanced expression levels of Nos2, Ccl8, and Cxcl9 for M1 and Arg1, Cxcl13, and Tgf-β for M2 (Supplemental Fig. 1G). These results indicated that aging caused significant alterations in lung-resident AMs.

**Fig. 2:**
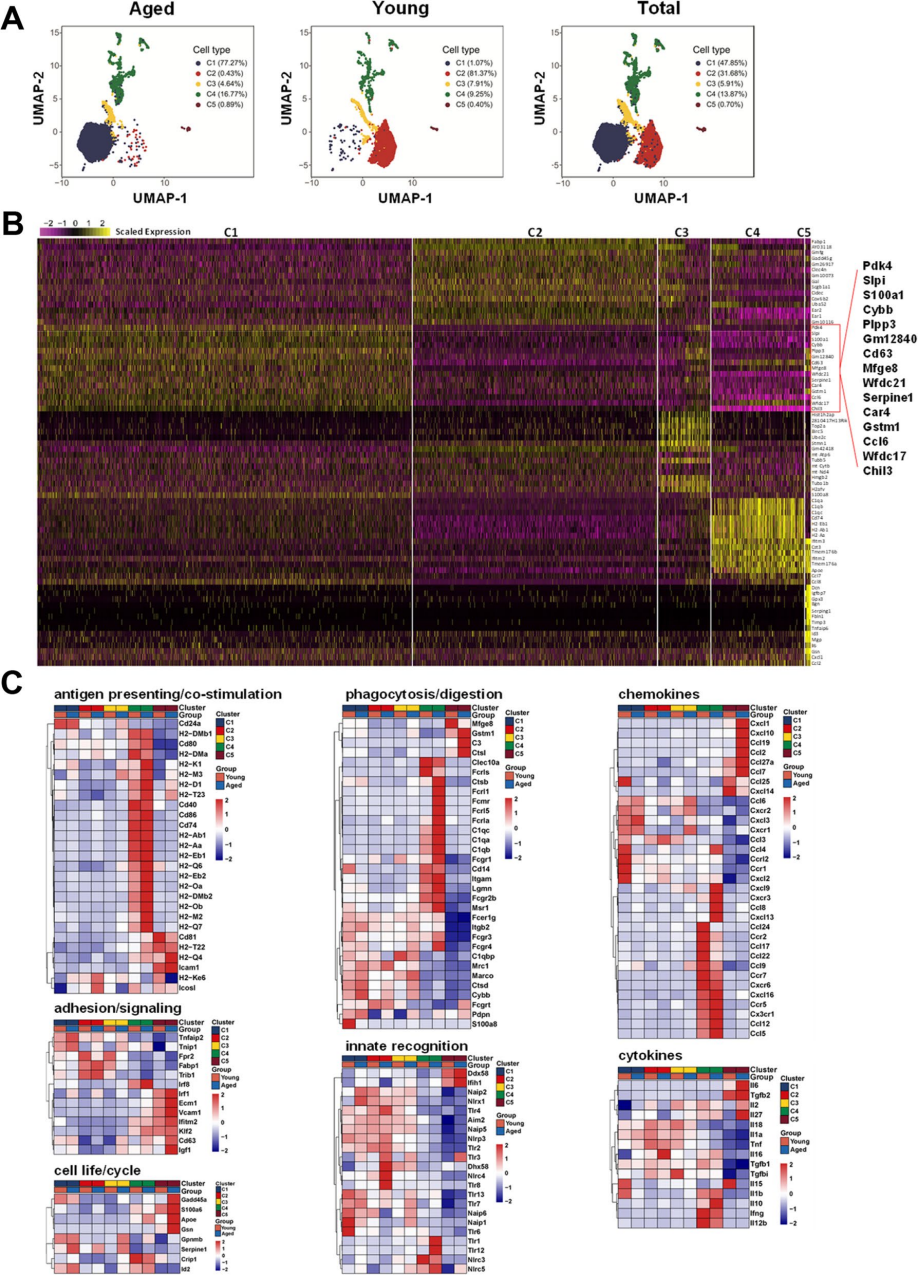
Single cell transcriptional profiling identified five AM populations in aged and young mice. AMs (CD45^+^ F4/80^+^ CD11c^+^) purified from lung MNCs (10 mice/sample) were analyzed through single cell RNA-seq. **A** Uniform manifold approximation and projection (UMAP) plots showed cell clustering based on gene expression. Five transcriptionally distinct clusters (C1-C5) were identified for the AMs in aged and young mice. **B** Heatmap showing the top 15 marker genes for each cluster (C1-C5). C1 marker genes are shown in larger font. **C** mRNA expression levels of functional molecules in each cluster (C1-C5) were compared between aged and young AMs

Furthermore, single-cell RNA sequencing was used to analyze aged AMs (12,622 among 12,952 cells, ≥ 200 genes per cell) and young AMs (7964 among 8163 cells, ≥ 200 genes per cell). Uniform manifold approximation and projection (UMAP) plots showed five distinct transcriptional distinct clusters C1-C5 (Fig. 2A). In the aged mice, the majority of AMs were in Cluster 1 (77.27%), with the remainder in Cluster 4 (16.77%) and Cluster 3 (4.64%), and few cells were in Cluster 2 (0.43%) and Cluster 5 (0.89%). In contrast, in the young mice, 81.37% of AMs were in Cluster 2, while almost all remainders were in Cluster 3 (7.91%) and Cluster 4 (9.25%), and few cells were in Cluster 1 (1.07%) and Cluster 5 (0.40%). These results showed that Cluster 2 essentially disappeared in aged mice, and was substituted by Cluster 1. The top 15 marker genes for each cluster showed that Cluster 1 was characterized by higher expression levels of Gstm1, Serpine1, Cybb, Pdk4 and Cd63, indicating their long survival time and enhanced cell senescence (Fig. 2B).

Compared to the young Cluster 2, aged Cluster 1 displayed higher expression levels of Cd24a, MARCO, Ctsd, Cybb, Pdpn, Cxcr1, Cxcr2, Cxcl3 and Ccl6, and lower expression levels of Fabp1, Fpr2 and Trib1 (Fig. 2C). Higher expression levels of Gpnmb, Serpine1 and Id2 showed aged AMs of Cluster 1 with a longer life compared with Cluster 2 (Fig. 2C). Furthermore, Cluster 4 displayed the highest expression levels of molecules for antigen presentation and co-stimulation, phagocytosis and digestion, and chemokines among these clusters in the aged, which was similar to the young (Fig. 2C). However, higher expression levels of Cxcr3, Ccl8, Cxcl13 and Cxcl9 were observed in Cluster 4 of the aged compared to the young (Fig. 2C). These results indicated that the newly emerged Cluster 1 and increased Cluster 4 were responsible for the alterations of AMs in the aged mice.

### Aged AMs are featured with terminal differentiation trajectories

When compared with Cluster 2 of young mice, Cluster 1 of aged mice showed significant differences in gene expression with 161 DEGs (supplementary Fig. 2A). The top 49 DEGs (36 upregulated genes and 13 downregulated genes) further demonstrated aged AMs in Cluster 2 had altered functions, such as producing several kinds of chemokines Ccl8, Ccl6, Cxcl3, Cxcl1, and Ccl9 (supplementary Fig. 2B). The DEGs were enriched in the pathways of cell migration, chemokine signaling, inflammatory response, and innate recognition signaling (supplementary Fig. 2C and D). These results further indicated that these two clusters were distinct cell subsets.

**Fig. 3:**
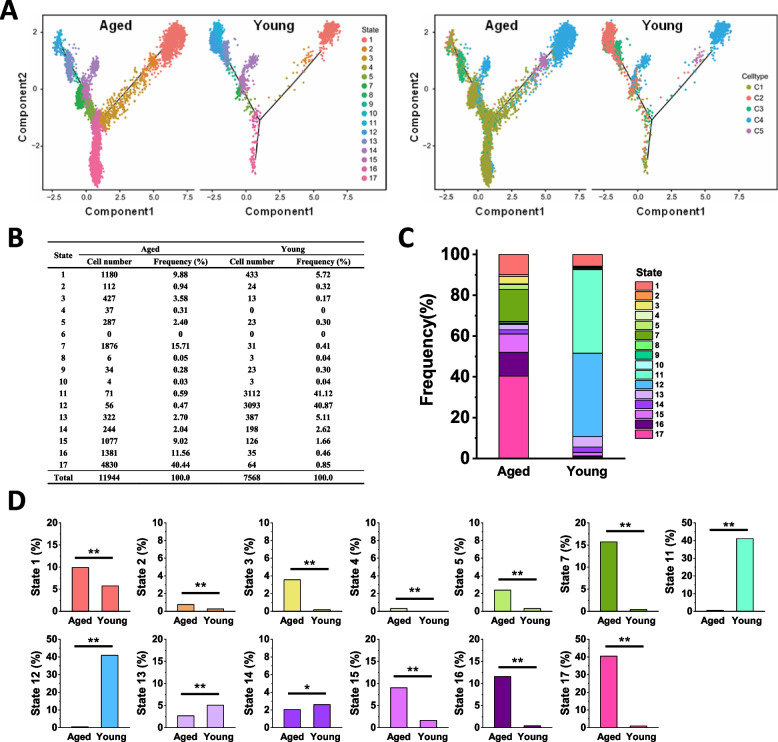
Aged AMs were featured with distinguished differentiation states compared with young AMs. Pseudotime trajectory analysis of AMs for the indicated groups. **A** Pseudotime trajectory analysis of AMs from the aged and young mice group (aged, *n* = 11,944; young, *n* = 7568) was conducted using Monocle 2. Components 1 and 2 were shown, and each dot represented a single cell. Dots were colored according to their state (left) and cell cluster (right). **B** Percentages and cell numbers of AMs in the indicated states were shown in a table. **C** Percentages of AMs in the indicated states were shown as a bar graph. **D** Statistical analysis of the percentages of AMs in the indicated states between the aged and young mice. A Chi-square test was used. *p < 0.05, **p < 0.01

Using pseudotime analysis, States 1 to 17 were identified for aged AMs and young AMs (Fig. 3A, left), which mainly resulted from Cluster 1 of aged AMs and Cluster 2 of young AMs (Fig. 3A, right). As shown in Fig. 3B and C, the AMs in Cluster 1 were distributed in State 17 (40.44%), State 16 (11.56%), State 15 (9.02%), and State 7 (15.71%), while the AMs in Cluster 2 were distributed in State 11 (41.12%) and State 12 (40.87%). There was no significant difference in the cell states for cluster 3 (mainly state 13), cluster 4 (mainly state 1 and state 14) and cluster 5 (state 2) between the aged and young groups (Fig. 3B and C). Aged AMs of Cluster 1 had distinct terminal differentiation trajectories, which were distinguished from young AMs of Cluster 2. In total, the differences in the frequency of cell numbers in each state showed that aged AMs in States 17, 16, 15 and 7 deserved further investigation, compared with young AMs in States 11 and 12 (Fig. 3D).

### A population of MARCO^+^ AMs with the ability to produce CCL6 are modulated by UGRP1 in the aging lungs

**Fig. 4:**
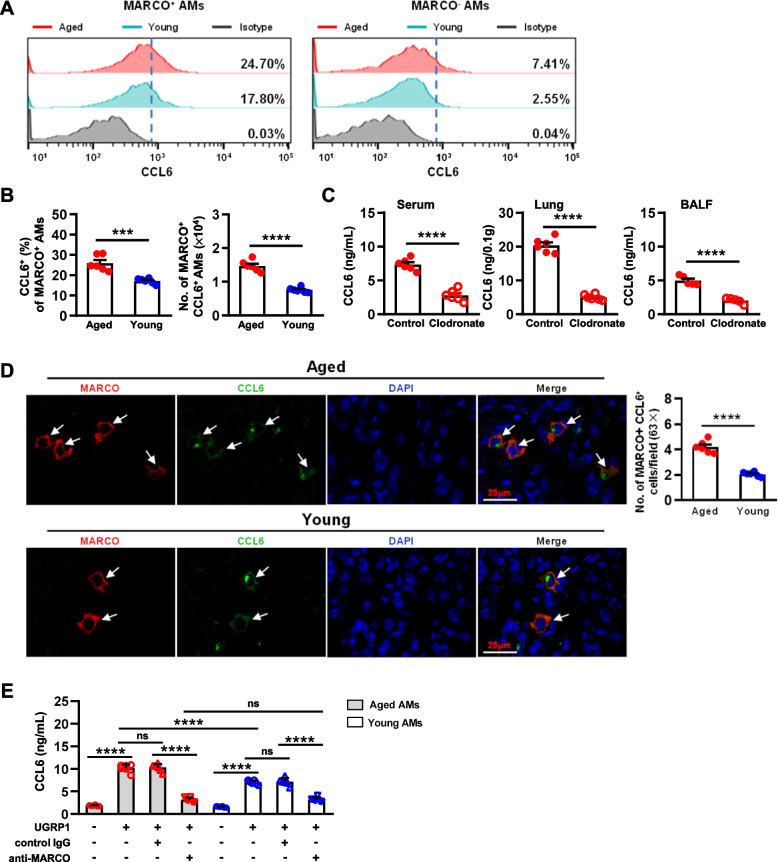
MARCO^+^ AMs produced CCL6 and were regulated by UGRP1 in aged lungs. The lung tissues of aged mice (20–24 months old) were compared with the young mice (10–16 weeks old). **A** Production of CCL6 in MARCO^+^ AMs and MARCO^−^ AMs. Lung MNCs were prepared and then analyzed by flow cytometry analysis. MARCO^+^AMs (CD45^+^ F4/80^+^ CD11c^+^) or MARCO^−^ AMs (CD45^+^ F4/80^+^ CD11c^+^) were gated to show the expression of CCL6. Histograms are shown respectively. **B** Frequency and absolute number of MARCO^+^ CCL6^+^ AMs in the aged lungs compared with the young lungs. **C** The levels of CCL6 in the serum, lung tissues (*n* = 6 for each group) and BALF (1.0 mL/mouse, *n* = 5 for each group) were detected by ELISA. Clodronate liposomes (50 μL/mouse) were administrated i.n. twice every 72 h to deplete AMs in the lungs. **D** Co-staining of MARCO and CCL6 in the lung tissues detected by immunofluorescence. The arrows indicate the positive cells in the lung tissues. Scale bar, 25 μm. MARCO^+^ CCL6^+^ cell numbers were counted and analyzed. Each symbol represents the average of 10 fields of vision (63 ×) from an individual sample. There were 6 mice in each group. Data are shown as the mean ± SEM. Comparisons by unpaired two-tailed Student’s *t*-test. **** *p* < 0.0001. (E) Purified AMs (CD45^+^ F4/80^+^ CD11c^+^) (1 × 10^5^ cells/well) were stimulated with UGRP1 (300 ng/mL) for 48 h in the DMEM containing 10% FBS, and then the levels of CCL6 in the culture supernatants were detected by ELISA. Anti-MARCO (20 μg/mL) was used to block the ligand-receptor interaction. There were 6 samples in each group. Data are shown as the mean ± SEM. Comparisons by two-way analysis of variance (ANOVA) followed by Tukey’s test. ns, not significant (p > 0.05), **** *p* < 0.0001

According to the marker genes for the predominant AMs in State17 (supplementary Table 4), the significantly increased mRNA expression levels of Mfge8, Gstm1, Ctsd, Gpnmb, Cd24a, Marco, and Ccl6 were shown for aged AMs compared with young AMs (supplementary Fig. 3A and C). Notably, increased cell numbers of MARCO^+^ AMs were observed (supplementary Fig. 3B). Furthermore, MARCO^+^AMs were gated to be analyzed by FCM (supplementary Fig. 3D) and aged MARCO^+^AMs exhibited a higher ability to produce CCL6 than the young, but not the MARCO^−^ AMs (Fig. 4A and B). The absolute number of MARCO^+^ CCL6^+^ AMs in the aged mice was significantly higher than that of the young mice (Fig. 4B). When AMs including MARCO^+^AMs were depleted by the clodronate liposomes treatment (supplemental Fig. 4A and B), markedly reduced levels of CCL6 were observed in the serum, lung tissues and broncho-alveolar lavage fluid (BALF) of aged mice (Fig. 4C). Markedly increased numbers of MARCO^+^ CCL6^+^ cells were observed in the aged lung tissue as shown by immunofluorescence (IF) staining (Fig. 4D), which further confirmed that MARCO^+^AMs were the main producer of CCL6 in the aged mice.

When stimulated in vitro by UGRP1, aged AMs produced considerably higher levels of CCL6 than young AMs (Fig. 4E). The MARCO blockade through anti-MARCO mAb treatment significantly inhibited the production of CCL6, confirming that the interaction between UGRP1 and its receptor MARCO accounted for the induction of CCL6 in the AMs (Fig. 4E). These results demonstrated that upregulated UGRP1 modulated the function of MARCO^+^ AMs with the stronger ability to produce CCL6, which was responsible for the higher levels of CCL6 in the aging lungs.

### UGRP1-modulated MARCO^+^ AMs promote pulmonary fibrosis in a CCL6-dependent manner

**Fig. 5:**
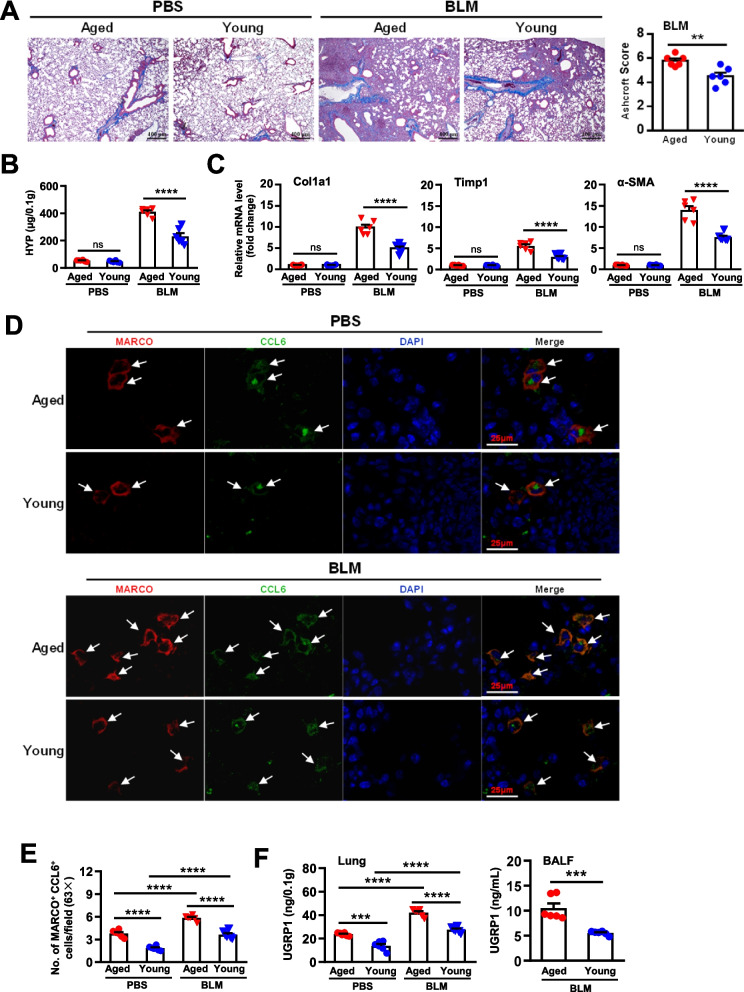
Aggravated lung fibrosis was related to increased accumulated MARCO^+^CCL6^+^ AMs in aged mice. Bleomyclin (BLM, 2.5 mg/kg, i.n.) was used to induce lung fibrosis in mice. Lung samples were harvested 21 days post-BLM treatment. **A** Histopathology of lung tissue was performed by Masson Trichrome staining. Ashcroft scores were used to indicate the degree of fibrosis. Data are shown as the mean ± SEM. Comparisons by unpaired two-tailed Student’s *t*-test. ** *p* < 0.01. **B** The hydroxyproline in lung tissue was detected by using hydroxyproline microplate assay kit. **C** The mRNA expression levels of Col1a1, Timp1 and α-SMA in lung tissue were detected by using real-time PCR. **D** Co-staining of MARCO and CCL6 in the aged lung tissues detected by immunofluorescence, compared with those in the young. The arrows indicate the positive cells in the lung tissues. Scale bar, 25 μm. **E** MARCO^+^ CCL6^+^ cell numbers were counted and analyzed. Each symbol represents the average of 10 fields of vision (63 ×) from an individual sample. **F** The levels of UGRP1 in the lung tissues and BALF (1.0 mL/mouse) were detected by ELISA. There were 6 mice in each group. Data are shown as the mean ± SEM. Comparisons by two-way analysis of variance (ANOVA) followed by Tukey’s test or by unpaired two-tailed Student’s *t*-test. ns, not significant, *** *p* < 0.001, **** *p* < 0.0001

To determine the role of MARCO^+^ AMs in vivo, a mouse model of lung fibrosis induced by intranasal administration of bleomycin (BLM, 2.5 mg/kg) was studied. BLM treatment led to epithelial cell death in the first 3 days, then induced excessive inflammation in days 3–9, and ultimately fibrosis with a peak around days 14–21 [19]. Pulmonary fibrosis was aggravated 21 days post-BLM treatment in the aged mice, as shown by the increased Masson staining and the higher Ashcroft score according to the distorted alveolar structure, thickened alveolar walls, and formation of fibrotic foci (Fig. 5A). The higher levels of hydroxyproline (HYP) were observed in the lung tissues of BLM-treated aged mice compared with BLM-treated young mice (Fig. 5B). Consistently, the mRNA expression levels of fibrogenic protein genes including Col1a1, Timp1 and α-SMA, were significantly upregulated in BLM-treated aged lungs compared with BLM-treated young lungs (Fig. 5C). Further, in the aggravated fibrotic lungs of aged mice, more MARCO^+^CCL6^+^ AMs were observed (Fig. 5D and E), indicating the importance of MARCO^+^ AMs and their production of CCL6 in the development of pulmonary fibrosis. Meanwhile, the levels of UGRP1 in the BLM-treated aged lungs were significantly higher than that of the young mice (Fig. 5F).

**Fig. 6:**
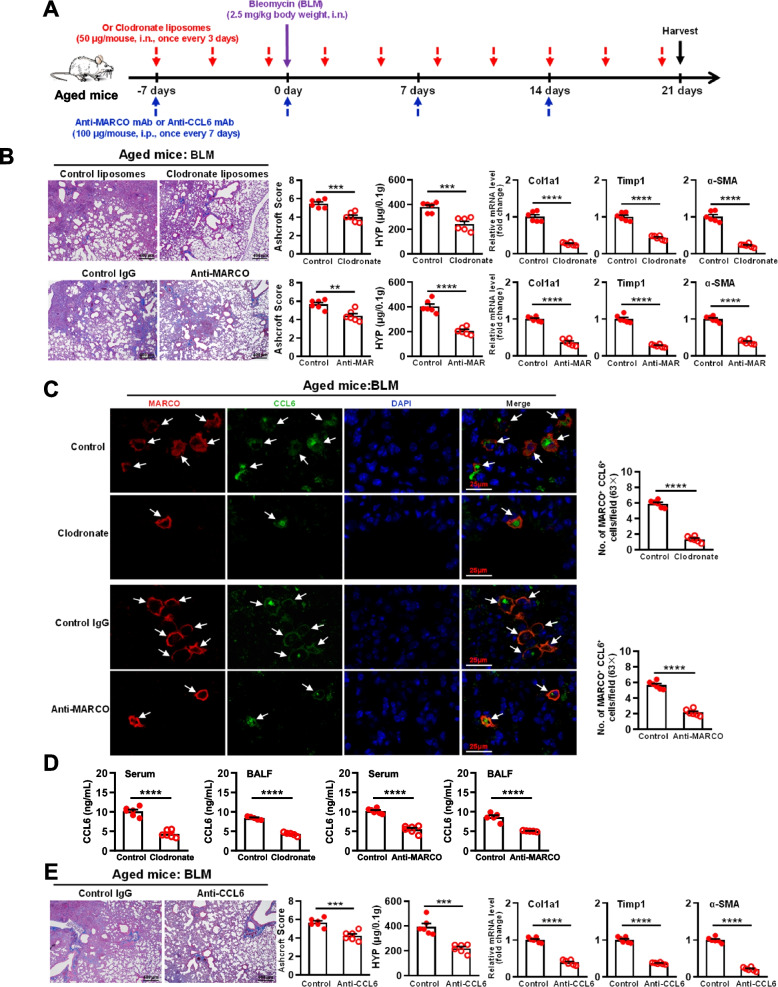
Targeting MARCO^+^ AMs significantly controlled lung fibrosis development by reducing CCL6 in the aged mice. **A** Experimental protocol for Clodronate liposomes, anti-MARCO or anti-CCL6 treatment. Bleomyclin (BLM, 2.5 mg/kg, i.n.) was used to induce lung fibrosis in mice. Clodronate liposomes (50 μL/mouse, one time per 3 days), anti-MARCO (100 μg/mouse, one time per 7 days) or anti-CCL6 mAb (100 μg/mouse, one time per 7 days) were used to treat the aged mice 7 days before BLM treatment. Lung and serum samples were harvested 21 days post-BLM treatment. **B** Histopathology of lung tissue by Masson Trichrome staining for Clodronate liposomes-treated and anti-MARCO-treated aged mice. Ashcroft scores were used to indicate the degree of fibrosis. Scale bar, 100 μm. The hydroxyproline in lung tissue was detected by using hydroxyproline microplate assay kit. The mRNA expression levels of Col1a1, Timp1 and α-SMA in lung tissue were detected by using real-time PCR. **C** Co-staining of MARCO and CCL6 in the aged lung tissues detected by immunofluorescence for clodronate liposomes-treated and anti-MARCO-treated aged mice, compared with the control. The arrows indicate the positive cells in the lung tissues. Scale bar, 25 μm. MARCO^+^ CCL6^+^ cell numbers were counted and analyzed. Each symbol represents the average of 10 fields of vision (63 ×) from an individual sample. There were 6 mice in each group. **D** The levels of CCL6 in the serum (*n* = 6 for each group) and BALF (1.0 mL/mouse, *n* = 5 for each group) were detected by ELISA. **E** Histopathology of lung tissue by Masson Trichrome staining for CCL6-neutralized aged mice. Ashcroft scores were used to indicate the degree of fibrosis. Scale bar, 100 μm. The hydroxyproline in lung tissue was detected by using hydroxyproline microplate assay kit. The mRNA expression levels of Col1a1, Timp1 and α-SMA in lung tissue were detected by using real-time PCR. There were 6 mice in each group. Data are shown as the mean ± SEM. Comparisons by unpaired two-tailed Student’s *t*-test. ** *p* < 0.01, *** *p* < 0.001, **** p < 0.0001

The depletion of MARCO^+^ AMs was performed by clodronate liposomes treatment (Fig. 6A, Supplementary Fig. 4B). This attenuated pulmonary fibrosis in aged mice shown by decreased Masson staining and Ashcroft scores, reduced tissue levels of HYP and reduced expression levels of fibrogenic protein genes including Col1a1, Timp1, and α-SMA, which was similar to the MARCO blockade by anti-MARCO treatment (Fig. 6A and B), further confirming the critical role of MARCO^+^AMs in promoting pulmonary fibrosis in aged mice. Depletion of AMs or blockade of MARCO caused a decrease in CCL6, as shown by the fewer CCL6^+^ cells in the lung tissue (Fig. 6C) and markedly lower levels of CCL6 in serum and BALF (Fig. 6D). CCL6 could be neutralized by anti-CCL6 mAb treatment (supplementary Fig. 4C). BLM-induced pulmonary fibrosis was significantly prevented in CCL6-neutralized aged mice, as shown by the decreased Masson staining and Ashcroft score, reduced tissue levels of HYP, and reduced expression levels of Col1a1, Timp1, and α-SMA (Fig. 6E). Additionally, treatment with UGRP1 protein significantly promoted the pulmonary fibrosis of young mice, as shown by the increased Masson staining, Ashcroft score, tissue levels of HYP and expression levels of Col1a1, Timp1 and α-SMA (supplementary Fig. 5A-C). Consistently, more accumulated MARCO^+^AMs and enhanced expression of CCL6 were induced by UGRP1 treatment (supplementary Fig. 5D-F). These results indicated that UGRP1-modulated-MARCO^+^AMs promoted pulmonary fibrosis dependent on CCL6 in aged mice.

### Age-related upregulation of UGRP1 and MARCO^+^ AMs observed in human lung tissues

**Fig. 7:**
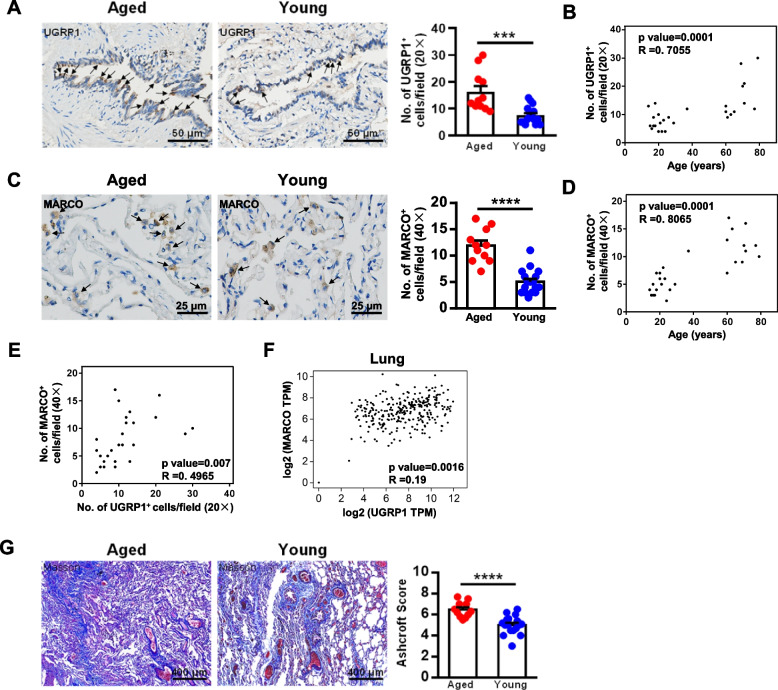
Age-related expression levels of hUGRP1 and hMARCO. Lung samples of nonsmoking aged patients with bullous lung disease (≥ 60 years old, *n* = 11) and nonsmoking young patients with bullous lung disease (≤ 40 years old, *n* = 17) were detected by immunohistochemistry. **A** Expression levels of human UGRP1. The arrows indicate the UGPR1 positive cells. Scale bar, 50 μm. UGPR1^+^ cell numbers were counted and analyzed. Each symbol represents the average of 10 fields of vision (20 ×) from an individual sample. **B** Correlation analysis of UGRP1^+^ cells and age (*n* = 28). **C** Expression levels of human MARCO. The arrows indicate the MARCO positive cells. Scale bar, 25 μm. MARCO^+^ cell numbers were counted and analyzed. Each symbol represents the average of 10 fields of vision (40 ×) from an individual sample. Scale bar, 25 μm. **D** Correlation analysis of MARCO^+^ cells and age (*n* = 28). **E** Correlation analysis of UGRP1^+^ cells to MARCO^+^ cells in the lung tissue samples (*n* = 28). **F** Correlation analysis of the gene expression of UGRP1 to MARCO in the lung tissues by GTEx data using GEPIA2 software. The Pearson correlation coefficient was calculated (**B**, **D**, **E** and **F**). **G** Lung samples were detected by Masson Trichrome staining. Ashcroft scores were used to indicate the degree of fibrosis. Data are shown as the mean ± SEM. Comparisons by unpaired two-tailed Student’s *t*-test. *** *p* < 0.001, **** *p* < 0.0001

To determine the expression of UGRP1 and MARCO^+^AMs in human lung tissues, lung samples were obtained from nonsmoking aged patients with bullous lung disease (≥ 60 years old, *n* = 11) and nonsmoking young patients with bullous lung disease (≤ 40 years old, *n* = 17). The expression of UGRP1 was significantly enhanced in aged lung tissue compared with young lung tissue (Fig. 7A), revealing a positive correlation with age (Fig. 7B). Consistently, a larger number of MARCO^+^ cells were observed in aged lung tissues and the number was positively correlated with age (Fig. 7C and D). The positive correlation of the expression of UGRP1 and MARCO was demonstrated by the data from aged patients and young patients (Fig. 7E), and data from the GTEx database further confirmed that the expression of UGRP1 positively correlated with expression of MARCO in the lung tissues (Fig. 7F). Aggravated Masson staining revealed the fibrosis in the aged lung tissues, as shown by the higher Ashcroft Score (Fig. 7G). These results showed that in humans, the expression levels of UGRP1 were age-related, and consequently modulated MARCO^+^AMs were involved in the progression of lung fibrosis.

## Discussion

It is estimated that the proportion of the world's population above the age of 60 years will represent 22% of the global population by 2050 [20]. Pulmonary diseases have significant consequences for the aging population, such as COPD and pulmonary fibrosis. Hence, exploring the aging process of the lungs is essential to provide optimal treatment for the elderly population. One of the hallmarks of aging is the progressive deterioration of immune functions [21]. Regarding the lung, numerous age-related alterations in the respiratory and pulmonary immune systems partly account for the higher risk for chronic pulmonary diseases [3, 20]. In this study, we demonstrated that in the aging lungs, the expression levels of UGRP1 were significantly upregulated, which modulated MARCO^+^AMs to produce high levels of CCL6, accounting for the susceptibility to pulmonary fibrosis. By neutralizing CCL6 or targeting the interaction of UGRP1-MARCO, pulmonary fibrosis could be markedly prevented. Our study provides reliable evidence and effective means for the prevention and treatment of chronic pulmonary disease in elderly individuals.

The term ‘inflammageing’ was coined to describe the age-related dysregulated persistent inflammation in the steady state in the aged [22]. Considerably enhanced expressions of proinflammatory cytokines (such as TNF-α and IL-6), surfactant proteins (such as SP-A and SP-D), lipids, and complement components were observed in the aged lungs with a relatively oxidized environment in mice and humans [23]. Conversely, the enhanced levels of IL-10, which was produced by mononuclear phagocytes, suppressed the innate pulmonary granuloma cytokine response including TNF-α, IL-6, CCL3, and CXCL2, and the innate IL-12/IFN-γ axis in the lungs of aged mice [24, 25]. In the aged lungs, we observed higher levels of CCL2/3/4/6/9/12, CXCL1/2/10/11/12/13, IL-1β and TNF-α compared with the young lungs (sFigure 6A and Fig. 1D and E). These inflammatory chemokines and cytokines were also upregulated after BLM treatment (sFigure 6A and Fig. 5E). In the aged lungs, enhanced mRNA expression levels of IL-10 and IL-1β were observed in AMs (supplementary Fig. 1G), and enhanced mRNA expression levels of CCL6 and CXCL3 were observed in aged AMs of Cluster 1 (Fig. 2C). Furthermore, increased MARCO^+^ CCL6^+^ AMs were demonstrated in the aged lungs compared with the young mice (Fig. 4D). When stimulated by UGRP1, aged MARCO^+^ AMs could produce much higher levels of CCL6 than young MARCO^+^ AMs (Fig. 4E), which further confirmed the inflammageing in lungs.

Using single-cell RNA sequencing, AMs were further identified as five clusters, representing five subpopulations with the indicated molecular characteristics and functions (Fig. 2). Compared to the young AMs, aged AMs displayed DEGs such as the antiviral genes Eosinophil cationic protein 1 and 2 (Ear1 and Ear2), and the genes Gpnmb and Mfge8, which was consistent with the observation reported by Ilias Angelidis et al. [2]. They reported that aging led to changes in cellular activity states across 30 cell populations in the lungs of aged mice, including several kinds of immune cells, such as neutrophils, monocytes, B cells, AMs, dendritic cells, and CD4^+^ T cells [2]. However, why aging leads to changes in these immune cells is not clear. In this study, we demonstrated that in the aged lungs, there was a subpopulation of MARCO^+^AMs with a specific development state (Fig. 3 and Supplementary Fig. 3), which was modulated by its specific ligand UGRP1 expressed by airway epithelial cells (Figs. 1 and 4E). In 2003, UGRP1 was first identified as a lung-specific ligand for the MARCO receptor of AMs [14]. Notably, the expression level of UGRP1 was significantly upregulated in the aged lungs of mice and humans (Figs. 1A-D and 7A, B). The T/EBP/NKX2.1 homeodomain transcription factor regulates Ugrp1 gene activity at the transcriptional level [26]. Additionally, UGRP1 expression was partly regulated by the local cytokine environment, as it could be induced by Th1 cytokines, but suppressed by proinflammatory cytokines such as IL-9, and Th2 cytokine such as IL-5 [27–29].

Resident AMs are dispensable for the development of fibrosis [30]. The secretion of proinflammatory and fibrotic mediators such as TNF-α, TGF-β, IL-10, CCL18, and Chitinases played critical roles at each of the key stages of the fibrotic process [31]. In the aged lungs, AMs developed mixed M1/M2 phenotypes when compared to the young AMs (supplementary Fig. 1G), which might polarize to a predominant phenotype depending on the certain circumstances. A profibrotic effect of the CX3CR1^+^ transitional macrophages localized to the fibrotic niche was demonstrated in BLM-induced lung fibrosis [32]. In aged mice, AMs of Cluster 4 exhibited a considerably high expression level of CX3CR1 (Fig. 2C). Whether they are responsible for the susceptibility to pulmonary fibrosis in aged mice or not requires further investigation. Higher levels of NLRP3 inflammasome activation were observed in aging AMs in response to BLM, which contributed to the development of BLM-induced pulmonary fibrosis in aged mice [33]. Our study demonstrated that MARCO^+^ AMs were necessary for the aggravated BLM-induced pulmonary fibrosis in aged mice (Figs. 5D-E and 6B-D). MARCO modulated the alternative activation of macrophages for their polarization of M2 and the fibrotic responses to lung injury, which was also required for the development of chrysotile-induced pulmonary fibrosis [34]. Aged MARCO^+^AMs showed a stronger ability to produce CCL6 (Fig. 4), and were indispensable for the progression of pulmonary fibrosis (Fig. 6C-E). MARCO acts as an initial signaling receptor that binds environmental particles or ligands on epithelial cells, leading to profibrotic effects of AMs. These findings indicate that MARCO is an effective therapeutic target to halt the progression of pulmonary fibrosis.

Recently, it was demonstrated that aging macrophages promoted the sequestration of glucose into glycogen via upregulated prostaglandin E2 (PGE2) signaling through its EP2 receptor, which reduced the glucose flux and mitochondrial respiration, and drove maladaptive proinflammatory responses [35]. Differences in fat/carbohydrate digestion and absorption, glycosaminoglycan biosynthesis, arginine and proline metabolism, alanine, aspartate and glutamate metabolism were observed in the aged AMs compared to young AMs (supplementary Fig. 1F and supplementary Fig. 2D). In aged macrophages, cell-autonomous NAD^+^ synthesis was suppressed, leading to innate immune dysfunction toward a proinflammatory activation state [36]. Thus, cellular metabolism plays a pivotal role in programming immune functions of AMs.

Herein, MARCO^+^AMs in the aged mice were first demonstrated to be the main producers of CCL6, which was modulated by its ligand in situ (Fig. 4). However, there is a limitation of the causal relationship between UGPR1, CCL6 production, and lung fibrosis in our study. Additional experiments using siRNA or other means to silence UGRP1 in airway epithelial cells deserve to be performed to show its effects on less CCL6 production from MARCO^+^ AM and less lung fibrosis. CCL6, also named as C10, is selectively produced by macrophages with a sharp divergence in the regulation from other chemokines, suggesting its distinct functions in the host defense [37]. CCL6 played a critical role in the lung fibrosis, as the neutralization of CCL6 attenuated BLM-induced pulmonary fibrosis [38]. Higher expression levels of CCL6 accounted for the enhanced susceptibility to pulmonary fibrosis in aged mice (Fig. 6). In vivo CCL6 promoting BLM-induced pulmonary fibrosis should be demonstrated, which would further confirm the conclusion. Murine CCL6 shares homology with human CCL23/CCL15, which perform the similar roles in lung diseases [39, 40]. The mechanisms by which CCL6 promotes the progression of pulmonary fibrosis were not clarified in our study. It was observed that when CCL6 was neutralized in BLM-induced lung fibrosis, the expression levels of CCL3, CXCL1, CXCL2 and IL-1β were significantly reduced, indicating the effects of CCL6 on these chemokines and cytokine (sFigure 6B). It was reported that CCL6 attracted macrophages, CD4^+^ T cells and eosinophils [38–40]. The mechanisms of higher levels of mCCL6 or its homology hCCL23/CCL25 involved in the progression of lung diseases should be further investigated. Additionally, a CCL6-dependent prometastatic activity of eosinophils was observed [41]. The relationship between the high expression level of CCL6 in the aged host and age-related cancer also deserves further study.

In conclusion, aging lung epithelial cells with intrinsic alternations modulate the functions of AMs and are involved in the chronic pulmonary fibrosis. Our study elucidates the underlying immunological mechanisms of the age-related lung fibrosis, which is key to establishing optimal targeting for the aging population.

## Materials and methods

### Mice

Female C57BL/6 mice were obtained from the Shanghai Experimental Center of the Chinese Science Academy (Shanghai, China). Young mice (10–16 weeks) and aged mice (20–24 months) were used. All mice were maintained under specific-pathogen-free and controlled conditions (22 °C, 55% humidity, and a 12-h day/night rhythm), in accordance with the Guide for the Care and Use of Laboratory Animals granted by University of Science and Technology of China.

### Isolation of lung mononuclear cells (MNCs)

As previously described [42], MNCs were isolated from the lungs via density gradient centrifugation using 40% and 70% Percoll solution (Gibco BRL, Grand Island, NY, USA).

### Purification of alveolar macrophages

Isolated lung MNCs were stained with fluorescein isothiocyanate (FITC)-conjugated anti-F4/80 (Clone BM8, eBioscience, San Diego, CA, USA), phycoerythrin (PE)-conjugated anti-CD11c (Clone N418, eBioscience, San Diego, CA, USA) and allophycocyanin/cyanine7 (APC-Cy7)-conjugated anti-CD45 (Clone 104, Biolegend, San Diego, CA, USA). Subsequently, alveolar macrophages (CD45^+^ F4/80^+^ CD11c^+^) were sorted using a FACS Aria II flow cytometer (Becton Dickinson, Franklin Lakes, NJ, USA). The purity of the separated cells was > 95%.

### mRNA sequencing

Total RNA was extracted from the purified alveolar macrophages (CD45^+^ F4/80^+^ CD11c^+^) using a miRNeasy Mini Kit (QIAGEN, GmBH, Germany). The mRNA sequencing was described in the supplemental materials and methods. Differentially expressed genes (DEGs) were analyzed by Gene Ontology (GO) and KEGG pathway analysis as described in the Supplemental Materials and Methods.

### Single-cell RNA sequencing and analysis

Single-cell barcode scRNA-seq libraries were generated for purified alveolar macrophages (CD45^+^ F4/80^+^ CD11c^+^) using Chromium Single Cell 3′ Library (V2) (10 × Genomics, Pleasanton, CA, USA). A HiSeq X Ten system (Illumina) was used to sequence sc-RNA libraries. Data were mapped to the mouse genome mm10 using Cell Ranger 2.1.1 (10 × Genomics). For further analysis, raw data were converted to a Seurat object using Seurat R v2.3.4. [43] or a CellDataSet object using monocle R 2.10.0 [44].

### Flow cytometry analysis

As previously described [45], for the surface phenotype assays, 1 × 10^6^ cells were blocked with 10 μL rat serum for 30 min at 4 °C and then stained with the indicated antibody for 30 min at 4 °C in the dark. For the intracellular cytokine assay, the cells were stimulated with PMA (Sigma, St Louis, MO, USA), monensin (Sigma, St Louis, MO, USA) and ionomycin (Calbiochem, San Diego, CA, USA) for 4 h. The cells were labeled for surface markers, fixed, permeabilized, and then labeled with the indicated intracellular antibody for 30 min at 4 °C in the dark. All data were acquired using a FACS Aria II flow cytometer (Becton Dickinson, Franklin Lakes, NJ, USA) and analyzed using FlowJo software version 10.0 (Treestar, Ashland, OR, USA). The monoclonal antibodies (mAb) used for FACS are shown in Supplemental Table 1.

### Quantitative real-time polymerase chain reaction (PCR)

Total RNA was extracted from purified alveolar macrophages (CD45^+^ F4/80^+^ CD11c^+^) using a miRNeasy Mini Kit (QIAGEN, Duesseldorf, Germany). Total RNA was extracted from the lung tissue using TRIzol reagent (Invitrogen, Carlsbad, CA, USA). The process was performed as described in the Supplemental Materials and Methods. Gene expression levels were quantified using the ΔΔCt method. Information on gene-specific primers is shown in Supplemental Table 2.

### Stimulation of alveolar macrophages in vitro

Purified alveolar macrophages (1 × 10^5^ cells/well) were stimulated with 300 ng/mL UGPR1 (LS-G56865-1, LifeSpan, Hamilton, OH, USA) in a total volume of 200 µL (DMEM supplemented with 10% fetal bovine serum) for 48 h. CCL6 in the culture supernatants was detected by an ELISA kit (EMCCL6, Thermo Scientific, Frederick, MD, USA). The anti-mMARCO antibody (Clone ED31, GeneTex, Alton Pkwy Irvine, CA, USA) was used to block the interaction at a concentration of 20 μg/mL in vitro. Immunoglobulin (Ig)G (clone HRPN, BioXcell, West Lebanon, NH, USA) was used as the control.

### Histological examination

For histological examination, mouse lung samples or human lung samples from nonsmoking patients with bullous lung disease were fixed in 10% neutral-buffered formalin and embedded in paraffin. Sections of 4 μm thickness were stained with anti-mUGRP1 antibody (Clone 381,707, R&D, Abingdon, UK), anti-hUGRP1 antibody (Clone EPR11463, Abcam, Cambridge, UK), or anti-hMARCO antibody (NBP2-39,004, Novus Biologicals, Littleton, CO, USA) for IHC. The DAB Peroxidase Substrate Kit (PV-6000, ZSGB-BIOTECH Co., Ltd, Beijing, China). The sections were photographed using an Olympus IX73 microscope (Olympus, Tokyo, Japan). For immunofluescence analysis, sections of 4 μm thickness were stained with anti-mCCL6 antibody (Clone EPR23475-105, Abcam, Cambridge, UK), anti-mMARCO antibody (Clone EPR22944-64, Abcam, Cambridge, UK), and Four Color Multiplex Fluorescent Immunostaining Kit (Anti rabbit, abs50028, Absin, Shanghai, China) were used. The sections were photographed using Leica TCS SP5 confocal microscope (Leica, Wetzlar, Germany).

### Western blotting

Western blotting was used to detect the protein expression levels of CCL6 and UGRP1 in the lung tissues of the aged mice compared with the young mice. The anti-CCL6 antibody (Clone 262,016, R&D, Abingdon, UK), anti-UGRP1 antibody (Clone 381,707, R&D, Abingdon, UK) and anti-β-actin antibody (Clone EPR21242, Abcam, Cambridge, UK) were used. The details were shown in the Supplemental Materials and Methods.

### Mouse pulmonary fibrosis model

Bleomycin (BLM) (Nippon Kayaku Co., Ltd, Takasaki-shi, Japan) was used to induce pulmonary fibrosis in mice [46]. Histochemical analysis was performed by Masson Trichrome staining to indicate the fibrosis. Ashcroft scores were used to indicate the degree of fibrosis [47]. The hydroxyproline in lung tissue was detected by using hydroxyproline microplate assay kit (abs580066, Absin, Shanghai, China). The mRNA expression levels of Col1a1, Timp1 and α-SMA in lung tissue were detected by using real-time PCR. There were six mice in each group.

### Antibody blockade and neutralization

The anti-mCCL6 mAb (Clone 262,016, R&D, Abingdon, UK) or anti-mMARCO mAb (Clone ED31, GeneTex, Alton Pkwy Irvine, CA, USA) was injected i.p. into the aged mice (100 μg/mouse in 100 μL of PBS) 7 days before bleomycin (Nippon Kayaku Co., Ltd, Takasaki-shi, Japan) challenge, and additional injections were performed every 7 days. Control mice were administrated equal amounts of control antibody Rat IgG2b (clone LTF-2; BioXcell, West Lebanon, NH, USA) or Rat IgG1 (clone HRPN, BioXcell, West Lebanon, NH, USA) respectively.

### UGRP1 protein treatment

Recombinant murine UGPR1 (LS-G56865-1, LifeSpan, Hamilton, OH, USA) was injected i.p. into young mice (15 μg/mouse in 100 μL of PBS) 7 days before bleomycin (Nippon Kayaku Co., Ltd, Takasaki-shi, Japan) challenge, and additional injections were performed every 7 days. Control mice were administrated 100 μL PBS solution.

### Depletion of alveolar macrophages

Clodronate liposomes (Liposoma, Amsterdam, NL) were administered intranasally (i.n.) into the recipient mouse (50 μL/mouse, once every 3 days for 28 days) to deplete alveolar macrophages in the bleomycin-treated mice, 7 days before bleomycin treatment. Control liposomes (Liposoma, Amsterdam, NL) were used for the control mice.

### Statistical analysis

All data are shown as the mean ± standard error of the mean (SEM). Differences between individual data were analyzed using Student’s *t-*test, and two-way analysis of variance (ANOVA) when appropriate. Additional comparisons of proportions were made using the chi-squared test. Pearson’s test was performed for the correlation analysis. A *p* value < 0.05 was considered statistically significant.

## Supplementary Information

**Additional file 1: Table 1. **The monoclonal antibodies for FACS. **Table 2. **The primers for each gene detected by real-time PCR. **Table 3. **Clinical characteristics of patients with bullous lung disease in this study. **Table 4. **The marker genes for the predominant AMs in State17. 

**Additional file 2: Figure 1. **Intrinsically alteredcell number and gene expression of aged AMs.

**Additional file 3: Figure 2. **Cluster 1 agedAMs distinguished from Cluster 2 young AMs.

**Additional file 4: Figure 3.**Representative DEGs of state 17 were shown.

**Additional file 5: Figure 4. **Depletion of AMsby clodronate liposomes treatment and neutralization of CCL6 by anti-CCL6treatment.

**Additional file 6: Figure 5.** Treatment of UGRP1 protein aggravated the BLM-induced pulmonaryfibrosis of the young mice. 

**Additional file 7: Figure 6. **Expressions of inflammatorychemokines and cytokines in BLM-induced lung fibrosis model of aged micecompared with young mice. 

## Data Availability

All data generated or analyzed during this study are included in this published article and its supplementary information files.
