# Supplementary material for: UGRP1-modulated MARCO+ alveolar macrophages contribute to age-related lung fibrosis
Source: Immun Ageing. 2023 Mar 18;20:14. doi: 10.1186/s12979-023-00338-8 (PMC10024420; doi:10.1186/s12979-023-00338-8)
Supplement: Supplementary file 1 — Additional file 1: Table 1. The monoclonal antibodies for FACS. Table 2. The primers for each gene detected by real-time PCR. Table 3. Clinical characteristics of patients with bullous lung disease in this study. Table 4. The marker genes for the predominant AMs in State17. [file 12979_2023_338_MOESM1_ESM.docx]

**UGRP1-modulated MARCO^+^ Alveolar Macrophages Contribute to Age-related Lung Fibrosis**

Yongyan Chen^1, 3*^, Xiaolei Hao^1, 3^, Ming Li^6^, Zhigang Tian^1, 3, 4^ Min Cheng^2, 3, 4, 5*^

^1^ Hefei National Laboratory for Physical Sciences at Microscale, the CAS Key Laboratory of Innate Immunity and Chronic Disease, School of Basic Medical Sciences, Division of Life Sciences and Medicine, University of Science and Technology of China, Hefei, 230027, China.

^2^ Department of Geriatrics, Gerontology Institute of Anhui Province, The First Affiliated Hospital of USTC, Division of Life Sciences and Medicine, University of Science and Technology of China, Hefei, 230001, China.

^3^ Institute of Immunology, University of Science and Technology of China, Hefei, 230027, China.

^4^ Cancer Immunotherapy Center, the First Affiliated Hospital of University of Science and Technology of China (Anhui Provincial Hospital), Hefei, 230001, China.

^5^ Anhui Provincial Key Laboratory of Tumor Immunotherapy and Nutrition Therapy, Hefei, 230001, China.

^6^ Department of Pathology, The Second Affiliated Hospital of Anhui Medical University, Hefei, Anhui 230601, China.

Address correspondence and reprint requests to Min Cheng or Yongyan Chen. Min Cheng, Department of Geriatrics, Gerontology Institute of Anhui Province, The First Affiliated Hospital of USTC, Division of Life Sciences and Medicine, University of Science and Technology of China, Hefei 230031, China. E-mail: [chengmin@ustc.edu.cn](mailto:chengmin@ustc.edu.cn). Yongyan Chen, School of Basic Medical Sciences, Division of Life Sciences and Medicine, University of Science and Technology of China, Hefei, China, Hefei 230027, China. E-mail: [yychen08@ustc.edu.cn](mailto:yychen08@ustc.edu.cn).

**Supplemental materials and methods**

**mRNA sequencing**

RNA quality was confirmed with a 2100 Bioanalyzer from Agilent. cDNA was synthesized and amplificated with the SMARTer Ultra Low RNA Kit (Clontech, Mountain View, CA, USA) following the manufacturer’s instruction. A TrueSeq DNA library preparation kit (Illumina, USA) was used to prepare the RNA sequencing library according to the manufacturer's protocol. Illumina paired-end 150 bp sequencing was performed on a HiSeq2500 apparatus (Illumina, USA). Sequencing raw reads were preprocessed by filtering out rRNA reads, sequencing adapters, short-fragment reads and other low-quality reads using Seqtk. Hisat2 (version:2.0.4) was used to map the cleaned reads to the mouse GRCm38.p4 (mm10) reference genome with two mismatches. After genome mapping, Stringtie (version:1.3.0) was run with a reference annotation to generate FPKM values (Fragments Per Kilobase of exon model per Million mapped reads) for known gene models. Differentially expressed genes were identified using edgeR. The p-value significance threshold in multiple tests was set by the false discovery rate (FDR).

**Gene Ontology (GO) and KEGG pathway analysis**

GO and Kyoto Encyclopedia of Genes and Genomes (KEGG) pathway analysis were performed as previously described (1, 2). Differentially expressed genes (DEGs) (fold change≥2) were converted to Entrez-IDs for GO and KEGG analysis with R 3.2.3 software using the library GO stats 2.34.0 (Author: Adrian Alexa, Jorg Rahnenfuhrer) and the R Bioconductor genome-wide mouse annotations from package org.Mm.eg.db (version 3.4.0) (Author: Marc Carlson). The results were ranked according to the p values (p<0.05).

**Quantitative real-time polymerase chain reaction (PCR)**

Total RNA was extracted from the purified alveolar macrophages (CD45^+^ F4/80^+^ CD11c^+^) using a miRNeasy Mini Kit (QIAGEN, Duesseldorf, Germany). Total RNA was extracted from the lung tissue using TRIzol reagent (Invitrogen, Carlsbad, CA, USA). mRNA was reverse transcribed into cDNA in a 60 μL reaction volume containing the following reagents: 4 μg of total mRNA, 5 μmol/L oligdT, 0.5 mmol/L dNTPs, 12 μL of 5×buffer, 10 mmol/L DTT, 120 units of RNase inhibitor, 600 units of M-MLV and distilled water (ultrapure, DNase and RNase free). The RT reaction was performed at 37°C for 50 min, followed by heating at 70°C for 15 min. The standard 50 μL volume reaction contained 25 μL 2×PCR buffer, 2 μL cDNA template, 0.4 μmol/L forward and reverse primers. Quantitative real-time PCR was performed using Roche Light Cycler 480 II (Roche Diagnostics, Germany). PCR reactions were performed using a total of 45 cycles consisting of a 15 s melt at 95°C, followed by a 30 s annealing at 60°C, 30 s extension at 72°C. Each sample was analyzed in triplicate for each target gene. Gene expression levels were quantified using the ΔΔCt method. Information on gene-specific primers is shown in Supplemental Table 2.

***Western blotting***

Lung tissues were lysed in cell lysis buffer (Beyotime, China) with a protease inhibitor cocktail (Complete Mini; Roche, Switzer-land) for 5 min in ice, and then centrifuged at 13,000 g for 5 min at 4°C. The concentration of total protein in the supernatant was measured by BCA Protein Assay Kit (Thermo Fisher Scientific, Waltham, MA, USA). After SDS-PAGE, proteins were transferred onto PVDF membranes (Millipore Corp., Billerica, USA), and incubated with anti-CCL6 antibody (Clone 262016, R&D, Abingdon, UK), anti-UGRP1 antibody (Clone 381707, R&D, Abingdon, UK) or anti-β-actin antibody (Clone EPR21242, Abcam, Cambridge, UK) over-night at 4°C. Membranes were washed with 0.1% (vol/vol) Tween 20 in TBS (pH 7.6) and incubated with a 1:2,500 dilution of horseradish peroxidase-conjugated anti-rat IgG (CST, Danvers, MA, USA) for 60 min at room temperature. Protein bands were visualized by ECL reaction (Pierce Biotechnology, Rockford, IL, USA).

**Supplemental Table 1. The monoclonal antibodies for FACS**

| **Fluorescein** | **Antibody** | **Clone number** | **Manufacturer** | **Isotype control** |
| --- | --- | --- | --- | --- |
| FITC | anti-F4/80 | BM8 | eBioscience | Rat IgG2α, κ |
| PE | anti-CD11c | N418 | eBioscience | ArH IgG |
| APC | anti-MARCO | 579511 | R&D | Rat IgG1 |
| APC | anti-CD24 | 30-F1 | Biolegend | Rat IgG2c, κ |
| Alexa Fluor 750 | anti-CCL6 | 262016 | R&D | Rat IgG2b |
| Alexa Fluor 750 | Rat IgG2b | 141945 | R&D | - |
| APC-cy7 | anti-CD45.2 | 104 | Biolegend | Ms IgG2a, κ |
| BV510 | anti-CD45.2 | 104 | Biolegend | Ms IgG2a, κ |

**Supplemental Table 2. The primers for each gene detected by real-time PCR**

| **Gene** | **Forward primer (5’-3’)** | **Reverse primer (5’-3’)** | **Amplicon**  **length (bp)** | **Ref** |
| --- | --- | --- | --- | --- |
| ***Col1a1*** | GAAACCCGAGGTATGCTTGA | GACCAGGAGGACCAGGAAGT | 275 | *This work* |
| ***Timp1*** | tatgcccacaagtcccagaa | actctccagtttgcaaggga | 210 | *(3)* |
| ***α-SMA*** | ACTACTGCCGAGCGTGAGAT | AAGGTAGACAGCGAAGCCAG | 433 | *(4)* |
| ***CCL2*** | CCAGCAAGATGATCCCAATG | TACGGGTCAACTTCACATTC | 249 | *This work* |
| ***CCL3*** | GATTCCACGCCAATTCATCG | AGGCATTCAGTTCCAGGTCA | 155 | *(5)* |
| ***CCL4*** | tttctcttacacctcccggc | agctgctcagttcaactcca | 181 | *(5)* |
| ***CCL6*** | tgccacacagatcccatgta | gatcttgggccttgcttcag | 168 | *This work* |
| ***CCL7*** | ACGCTTCTGTGCCTGCTGCTC | CCTCGACCCACTTCTGATGG | 232 | *This work* |
| ***CCL9*** | tgccctctccttcctcattc | gataaagatgatgcccggcc | 247 | *This work* |
| ***CCL12*** | gaagattcacgtccggaagc | gggtcagcacagatctcctt | 111 | *This work* |
| ***CXCL1*** | GCACCCAAACCGAAGTCATA | AGAAGCCAGCGTTCACCAGA | 171 | *This work* |
| ***CXCL2*** | GCCCAGACAGAAGTCATAGC | CCTCCTTTCCAGGTCAGTTA | 130 | *This work* |
| ***CXCL10*** | TCATCCCTGCGAGCCTATCC | TGCGTGGCTTCACTCCAGTT | 167 | *This work* |
| ***CXCL11*** | tggcagagatcgagaaagct | acgttcccaggatgtcacat | 193 | *This work* |
| ***CXCL12*** | CAGAGCCAACGTCAAGCATC | CCACTTTAATTTCGGGTCAAT | 115 | *This work* |
| ***CXCL13*** | GTTGTCGGTCTAAACATCATAG | AATTCAGAGCAGGGATAAAA | 218 | *This work* |
| ***IL-1β*** | actcattgtggctgtggaga | ttgttcatctcggagcctgt | 199 | *(6)* |
| ***IL-6*** | aacgatgatgcacttgcaga | GGAAATTGGGGTAGGAAGGA | 276 | *(7)* |
| ***TNF-α*** | ccacatctccctccagaaaa | agggtctgggccatagaact | 259 | *(8)* |
| ***TGF-β1*** | attcagcgctcactgctctt | tctctgtggagctgaagcaa | 218 | *This work* |
| ***β-actin*** | TGACGTTGACATCCGTAAAGACC | CTCAGGAGGAGCAATGATCTTGA | 148 | (9) |

**Supplemental Table 3. Clinical characteristics** **of patients with bullous lung disease in this study**

|  | **Young** | **Aged** | **P value** |
| --- | --- | --- | --- |
| **Age**  **mean (min-max)** | 20.76 (14-37) | 67.81 (60-79) |  |
| **Sex (M/F)** | 16/1 | 9/2 | 0.304 |
| **Location of Bullae** |  |  | 0.993 |
| **left lung** | 9 | 6 |  |
| **right lung** | 5 | 3 |  |
| **bilateral lungs** | 3 | 2 |  |
| **Number of Bullae** |  |  | 0.954 |
| **single** | 6 | 4 |  |
| **multiple (≥2)** | 11 | 7 |  |
| **Total number** | 17 | 11 |  |

**Supplemental Table 4. The marker genes for the predominant AMs in State17**

| **No.** | **Gene** | **Fold Change** | **P value (adj)** | **No.** | **Gene** | **Fold Change** | **P value (adj)** |
| --- | --- | --- | --- | --- | --- | --- | --- |
| 1 | Mfge8 | 7.2883 | 0 | 43 | Tnfaip2 | 2.1571 | 1.32E-188 |
| 2 | S100a1 | 6.2603 | 0 | 44 | Spp1 | 2.1502 | 1.11E-78 |
| 3 | Plpp3 | 5.7978 | 0 | 45 | mt-Co2 | 2.1482 | 1.71E-183 |
| 4 | Gstm1 | 5.2582 | 0 | 46 | Itgax | 2.1252 | 5.49E-188 |
| 5 | Cd63 | 5.0882 | 0 | 47 | Sypl | 2.1247 | 4.01E-145 |
| 6 | Wfdc17 | 4.9981 | 0 | 48 | Myo5a | 2.1042 | 2.38E-205 |
| 7 | Gpnmb | 4.6356 | 0 | 49 | Atp6ap1 | 2.0925 | 4.77E-156 |
| 8 | Pdk4 | 4.6333 | 0 | 50 | Lpl | 2.0915 | 3.54E-195 |
| 9 | Psap | 4.1821 | 0 | 51 | Pld3 | 2.0698 | 1.27E-232 |
| 10 | Cybb | 4.0355 | 0 | 52 | Ncf1 | 2.0572 | 1.25E-125 |
| 11 | Sdc3 | 4.0020 | 0 | 53 | Hk2 | 2.0553 | 7.56E-106 |
| 12 | Fabp4 | 3.7211 | 0 | 54 | Igf1 | 2.0530 | 3.96E-149 |
| 13 | Ctsd | 3.6973 | 0 | 55 | mt-Atp6 | 2.0376 | 8.70E-122 |
| 14 | mt-Co1 | 3.4609 | 0 | 56 | Thbd | 2.0234 | 6.94E-91 |
| 15 | Marco | 3.3807 | 7.01E-93 | 57 | B3gnt7 | 2.0146 | 1.26E-125 |
| 16 | Cd200 | 3.3411 | 0 | 58 | Hsp90b1 | 2.0144 | 3.86E-142 |
| 17 | Ctsk | 3.3265 | 0 | 59 | Hebp1 | 2.0130 | 4.77E-176 |
| 18 | Fabp5 | 3.1262 | 0 | 60 | H2-K1 | 2.0106 | 3.96E-233 |
| 19 | S100a8 | 3.0767 | 0 | 61 | Gng12 | 2.0073 | 1.77E-146 |
| 20 | Slpi | 2.9926 | 0 | 62 | Gadd45a | 2.0004 | 2.14E-94 |
| 21 | Chil3 | 2.9797 | 0 | 63 | Lamp1 | 1.9893 | 8.66E-257 |
| 22 | Cxcr1 | 2.8494 | 0 | 64 | Mpeg1 | 1.9889 | 7.08E-215 |
| 23 | Pla2g2d | 2.7934 | 0 | 65 | Tpp1 | 1.9832 | 7.89E-115 |
| 24 | Slc39a2 | 2.6718 | 1.18E-202 | 66 | Cd36 | 1.9516 | 2.74E-85 |
| 25 | Cd24a | 2.6008 | 3.31E-218 | 67 | Crip1 | 1.9331 | 5.47E-205 |
| 26 | Ccl6 | 2.5903 | 0 | 68 | AC168977.1 | 1.9185 | 3.66E-173 |
| 27 | Serpine1 | 2.5892 | 8.94E-224 | 69 | Prkcd | 1.9156 | 1.25E-109 |
| 28 | Olr1 | 2.5882 | 1.17E-212 | 70 | Tkt | 1.9155 | 1.27E-137 |
| 29 | Ctsl | 2.5563 | 1.27E-263 | 71 | Tgfbr2 | 1.9121 | 4.84E-109 |
| 30 | Grn | 2.5499 | 0 | 72 | Ly75 | 1.9088 | 3.42E-101 |
| 31 | Creg1 | 2.5481 | 5.06E-288 | 73 | Mgll | 1.9078 | 2.76E-90 |
| 32 | Rgcc | 2.5476 | 4.50E-154 | 74 | mt-Cytb | 1.8892 | 1.49E-82 |
| 33 | Aldh2 | 2.5112 | 5.59E-206 | 75 | C77080 | 1.8597 | 3.33E-103 |
| 34 | Gns | 2.4512 | 6.07E-228 | 76 | Fosb | 1.8595 | 1.28E-95 |
| 35 | Serpinb6a | 2.3944 | 9.40E-217 | 77 | Lasp1 | 1.8535 | 1.45E-80 |
| 36 | mt-Nd2 | 2.3904 | 2.41E-187 | 78 | Cd84 | 1.8277 | 6.10E-90 |
| 37 | Car4 | 2.3053 | 9.99E-175 | 79 | Sdcbp | 1.8232 | 2.53E-181 |
| 38 | Bcap31 | 2.2744 | 1.18E-115 | 80 | Atp6v1c1 | 1.8151 | 3.54E-99 |
| 39 | mt-Nd4 | 2.2370 | 1.66E-140 | 81 | Gm12840 | 1.8118 | 2.00E-121 |
| 40 | Aldoc | 2.2316 | 5.40E-144 | 82 | Soat1 | 1.7898 | 3.00E-85 |
| 41 | Vat1 | 2.2129 | 2.81E-187 | 83 | Lipa | 1.7816 | 4.17E-92 |
| 42 | Wfdc21 | 2.1693 | 7.45E-163 | 84 | Bst1 | 1.7798 | 2.40E-82 |

**Supplemental Figure Legends**

**Supplemental Figure 1. Intrinsically altered cell number and gene expression of aged AMs.** The aged mice (20-24 months old) were compared with the young mice (10-16 weeks old). The lung MNCs were gated by FSC and SSC and analyzed by FCM. (A) Frequencies and numbers of AMs (CD45^+^ F4/80^+^ CD11c^+^). In the Aged-co group, the aged mice were co-housed with the young mice for 4 weeks. There were 6 mice for each group. The data are shown as the mean ± SEM. One-way analysis of variance (ANOVA) was used. ns, not significant (p > 0.05), ***p < 0.001, ****p < 0.0001. (B) Purification of AMs. AMs (CD45^+^ F4/80^+^ CD11c^+^) were purified from young and aged lung MNCs (20 mice/sample) by FACS respectively. (C) Purified AMs were analyzed through mRNA sequencing. The Volcano plots based on the fold-change and p value showed the differential expression of the indicated genes. The two vertical lines correspond to a two-fold change in the expression. The horizontal line indicates p = 0.05. Red plots represent the up-regulated genes. Blue plots represent the down-regulated genes. (D) Pie chart showing the distribution of DEGs in aged mice compared with that of young mice. (E&F) Biological pathways of DEGs. The list of DEGs was converted into Entrez-IDs for GO and KEGG analysis with R 3.2.3 using the library GOstats 2.34.0 and the R Bioconductor genomewide mouse annotations from the package org.Mm.eg.db (version 3.3.0). (G) Polarization to M1 and M2 for AMs of the aged mice compared with the young mice by mRNA sequencing analysis. AMs: alveolar macrophages; MNCs: mononuclear cells; FCM: flow cytometry; FACS: fluorescence activated cell sorting; DEGs: differentially expressed genes; FSC: forward scatter; SSC: side scatter; SEM: standard error of the mean; GO: Gene Ontology; KEGG: Kyoto Encyclopedia of Genes and Genomes.

**Supplemental Figure 2. Cluster 1 aged AMs distinguished from Cluster 2 young AMs.** Five transcriptionally distinct clusters (C1-C5) were identified for the AMs (CD45^+^ F4/80^+^ CD11c^+^) in aged and young mice through single cell RNA-seq (Figure 2). The gene expressions were compared between C1 and C2. (A) The Volcano plots based on the fold-change and p value showing the differential expression of the indicated genes. The two vertical lines correspond to a 1.2-fold change in expression. The horizontal line indicates p = 0.05. Red plots represent the up-regulated genes. Blue plots represent the down-regulated genes. (B) Heatmap showing the top 49 DEGs. Random 25 cell samples were shown in each cluster. (C&D) The list of DEGs was converted into Entrez-IDs for GO and KEGG analyses with R 3.2.3 using the library GOstats 2.34.0 and the R Bioconductor genomewide mouse annotations from the package org.Mm.eg.db (version 3.3.0). These DEGs were enriched in 30 biological pathways by GO enrichment analysis and significantly enriched in 25 pathways by the KEGG enrichment analysis. GO: Gene Ontology; KEGG: Kyoto Encyclopedia of Genes and Genomes.

**Supplemental Figure 3. Representative DEGs of state 17 were shown.** (A) Expressions of the state 17 representative marker genes were shown for AMs between aged and young mice. (B) The number of CD24^+^ AMs and MARCO^+^ AMs. The lung MNCs were counted and analyzed by FCM, and then the numbers of AM (CD45^+^ F4/80^+^ CD11c^+^) subpopulations were calculated according to the percentages. (C) Expressions of the state 17 representative marker gene *Ccl6* were shown. The mRNA expression levels of *Ccl6* in AMs were detected by single cell RNA-seq (left) and real-time PCR (right). There were 6 samples in each group for real-time PCR. The data are shown as the mean ± SEM. Student’s *t-*test was used. ns, not significant (p > 0.05), **p < 0.01, ****p < 0.0001. (D) The gating strategy of MARCO^+^ AMs and MARCO^-^ AMs for the intracellular CCL6 analysis. FCM: flow cytometry. AMs: alveolar macrophages.

**Supplemental Figure 4. Depletion of AMs by clodronate liposomes treatment and neutralization of CCL6 by anti-CCL6 treatment.** (A) Depletion of AMs. Clodronate liposomes (50 μL/mouse) were administrated i.n. to deplete AMs in the lungs of aged mice. After 3 days of administration, the depletion of AMs (CD45^+^ F4/80^+^ CD11c^+^) was confirmed by FCM analysis. (B) MARCO^+^ cells in the BLM-treated aged lungs after depleting AMs. Clodronate liposomes (50 μL/mouse, once every 3 days) was used to deplete AMs in the lung tissues 7 days before bleomyclin (BLM, 2.5 mg/kg, i.n.) treatment. The expression of MARCO in the aged lung tissues were detected by immunohistochemistry 21 days post-BLM treatment. The arrows indicate the positive cells in the lung tissues. Scale bar, 25 μm. MARCO^+^ cell numbers were counted and analyzed. Each symbol represents the average of 10 fields of vision (40×) from an individual sample. (C) Neutralization of CCL6 in the BLM-treated aged mice. Anti-CCL6 (100 μg/mouse, once every 7 days) was used to neutralize CCL6. The serum levels of CCL6 were detected by ELISA 21 days post-BLM treatment. There were 6 samples in each group. Data are shown as the mean ± SEM. Student’s *t-*test was used. ****p < 0.0001.

**Supplemental Figure 5.** **Treatment of UGRP1 protein aggravated the BLM-induced pulmonary fibrosis of the young mice.** Yong mice (10 weeks old) were treated with UGRP1 (15 μg/mouse, i.p., once every 7 days) 7 days before bleomyclin treatment (BLM, 2.5 mg/kg, i.n.). The histopathology of lung tissue was analyzed 21 days post-BLM treatment. (A) Masson Trichrome staining were shown and Ashcroft scores were used to indicate the degree of fibrosis. (B) The hydroxyproline in lung tissue was detected by using hydroxyproline microplate assay kit. (C) The mRNA expression levels of Col1a1, Timp1 and α-SMA in lung tissue were detected by using real-time PCR. (D) Co-staining of MARCO and CCL6 in the lung tissues detected by immunofluorescence for UGRP1-treated mice compared with the control. The arrows indicate the positive cells in the lung tissues. Scale bar, 25 μm. (E) MARCO^+^ CCL6^+^ cell numbers were counted and analyzed. Each symbol represents the average of 10 fields of vision (63×) from an individual sample. There were 6 mice in each group. (F) The serum levels of CCL6 were detected by ELISA. There were 6 mice in each group. Data are shown as the mean ± SEM. Student’s *t-*test or two-way ANOVA followed by Tukey’s test was used. ns, not significant (p > 0.05), **p < 0.01, *** p < 0.001, ****p < 0.0001.

**Supplemental Figure 6. Expressions of inflammatory chemokines and cytokines in BLM-induced lung fibrosis model of aged mice compared with young mice.** Bleomyclin (BLM, 2.5 mg/kg, i.n.) was used to induce lung fibrosis in mice. Lung samples were harvested 21 days post-BLM treatment. The aged mice (20-24 months old) were compared with the young mice (10-16 weeks old). (A) The mRNA expression levels of CCL2/3/4/7/9/12, CXCL1/2/10/11/12/13, IL-1β, IL-6, TNF-α and TGF-β1 in lung tissue were detected by using real-time PCR. (B) Anti-CCL6 mAb (100 μg/mouse, one time per 7 days) were used to treat the aged mice 7 days before BLM treatment. The mRNA expression levels of these inflammatory chemokines and cytokines were compared between anti-CCL6-treated aged mice and the control IgG-treated aged mice. There were 6 mice in each group. Comparisons by two-way analysis of variance (ANOVA) followed by Tukey’s test or unpaired two-tailed Student’s *t*-test. ns, not significant (p > 0.05), *p < 0.05, **p < 0.01, *** p < 0.001, ****p < 0.0001.

**Reference**

1. Ashburner M, Ball CA, Blake JA, Botstein D, Butler H, Cherry JM, et al. Gene ontology: tool for the unification of biology. The Gene Ontology Consortium. *Nat Genet.* 2000;25(1):25-9.

2. Kanehisa M, and Goto S. KEGG: kyoto encyclopedia of genes and genomes. *Nucleic acids research.* 2000;28(1):27-30.

3. Cheng M, Chen Y, Wang L, Chen W, Yang L, Shen G, et al. Commensal microbiota maintains alveolar macrophages with a low level of CCL24 production to generate anti-metastatic tumor activity. *Sci Rep.* 2017;7(1):7471.

4. Donkers JM, Roscam Abbing RLP, van Weeghel M, Levels JHM, Boelen A, Schinkel AH, et al. Inhibition of Hepatic Bile Acid Uptake by Myrcludex B Promotes Glucagon-Like Peptide-1 Release and Reduces Obesity. *Cell Mol Gastroenterol Hepatol.* 2020;10(3):451-66.

5. Liu L, Wang P, Wang YS, Zhang YN, Li C, Yang ZY, et al. MiR-130a-3p Alleviates Liver Fibrosis by Suppressing HSCs Activation and Skewing Macrophage to Ly6C(lo) Phenotype. *Front Immunol.* 2021;12:696069.

6. Yang Y, Cai X, Yang J, Sun X, Hu C, Yan Z, et al. Chemoprevention of dietary digitoflavone on colitis-associated colon tumorigenesis through inducing Nrf2 signaling pathway and inhibition of inflammation. *Mol Cancer.* 2014;13:48.

7. Chae MJ, Sung HY, Kim EH, Lee M, Kwak H, Chae CH, et al. Chemical inhibitors destabilize HuR binding to the AU-rich element of TNF-alpha mRNA. *Exp Mol Med.* 2009;41(11):824-31.

8. Llobet E, Martinez-Moliner V, Moranta D, Dahlstrom KM, Regueiro V, Tomas A, et al. Deciphering tissue-induced Klebsiella pneumoniae lipid A structure. *Proc Natl Acad Sci U S A.* 2015;112(46):E6369-78.

9. Mathew R, Futterweit S, Valderrama E, Tarectecan AA, Bylander JE, Bond JS, et al. Meprin-alpha in chronic diabetic nephropathy: interaction with the renin-angiotensin axis. *Am J Physiol Renal Physiol.* 2005;289(4):F911-21.
