# Supplementary figures and images for: UGRP1-modulated MARCO+ alveolar macrophages contribute to age-related lung fibrosis
Source: Immun Ageing. 2023 Mar 18;20:14. doi: 10.1186/s12979-023-00338-8 (PMC10024420; doi:10.1186/s12979-023-00338-8)

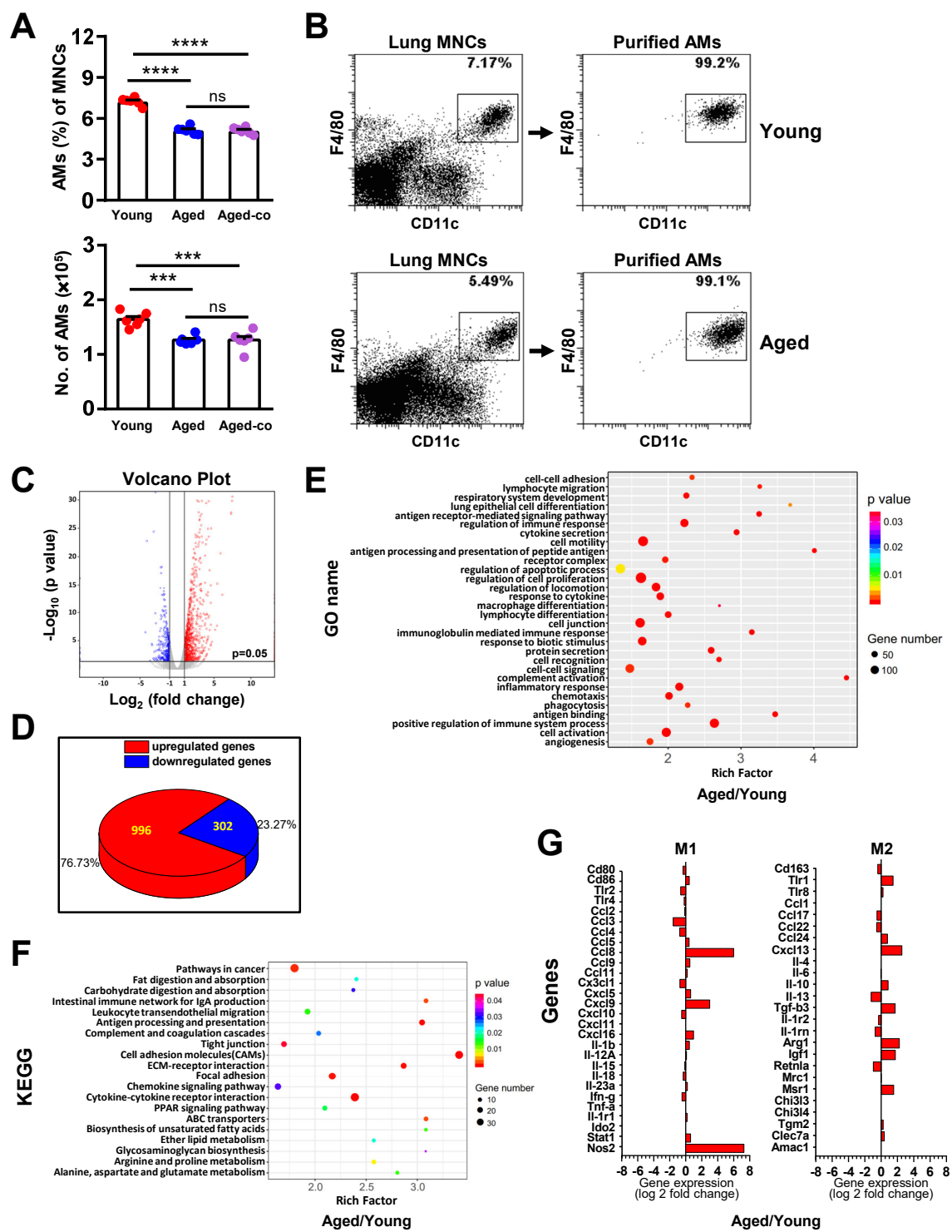

Supplement: Supplementary file 2 — Additional file 2: Figure 1. Intrinsically alteredcell number and gene expression of aged AMs. [file 12979_2023_338_MOESM2_ESM.pdf]

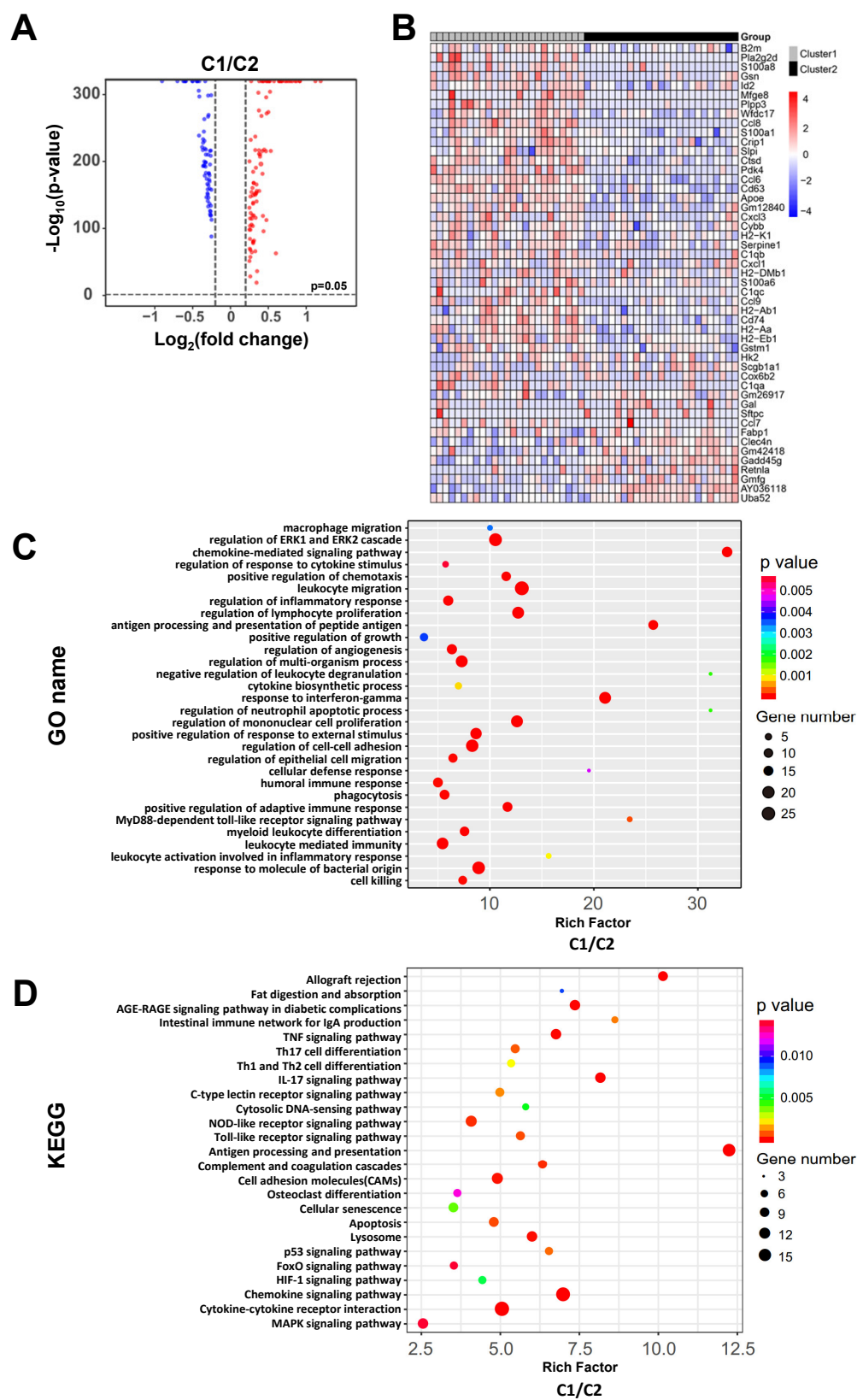

Supplement: Supplementary file 3 — Additional file 3: Figure 2. Cluster 1 agedAMs distinguished from Cluster 2 young AMs. [file 12979_2023_338_MOESM3_ESM.pdf]

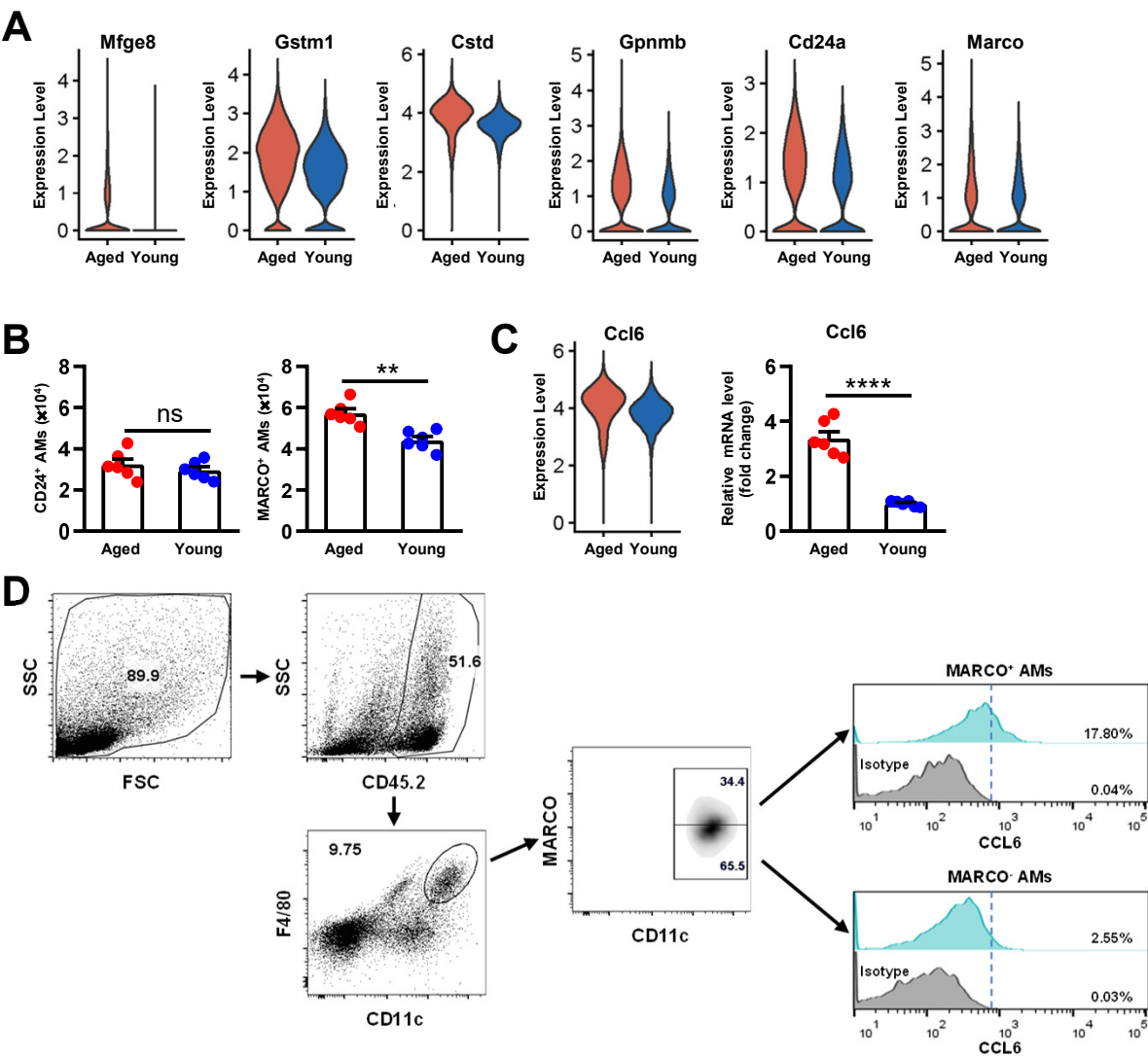

Supplement: Supplementary file 4 — Additional file 4: Figure 3.Representative DEGs of state 17 were shown. [file 12979_2023_338_MOESM4_ESM.pdf]

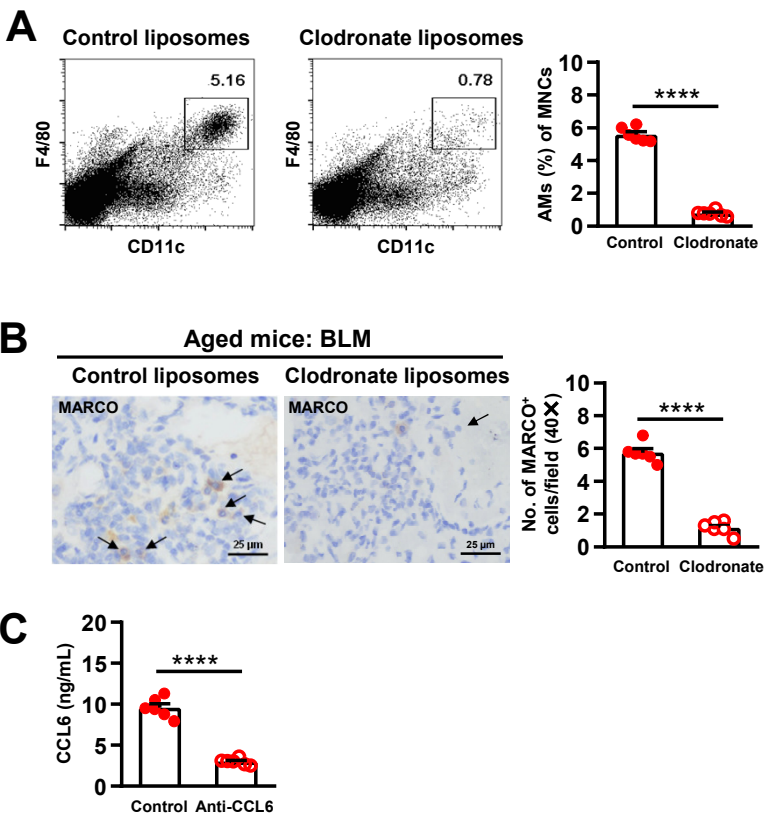

Supplement: Supplementary file 5 — Additional file 5: Figure 4. Depletion of AMsby clodronate liposomes treatment and neutralization of CCL6 by anti-CCL6treatment. [file 12979_2023_338_MOESM5_ESM.pdf]

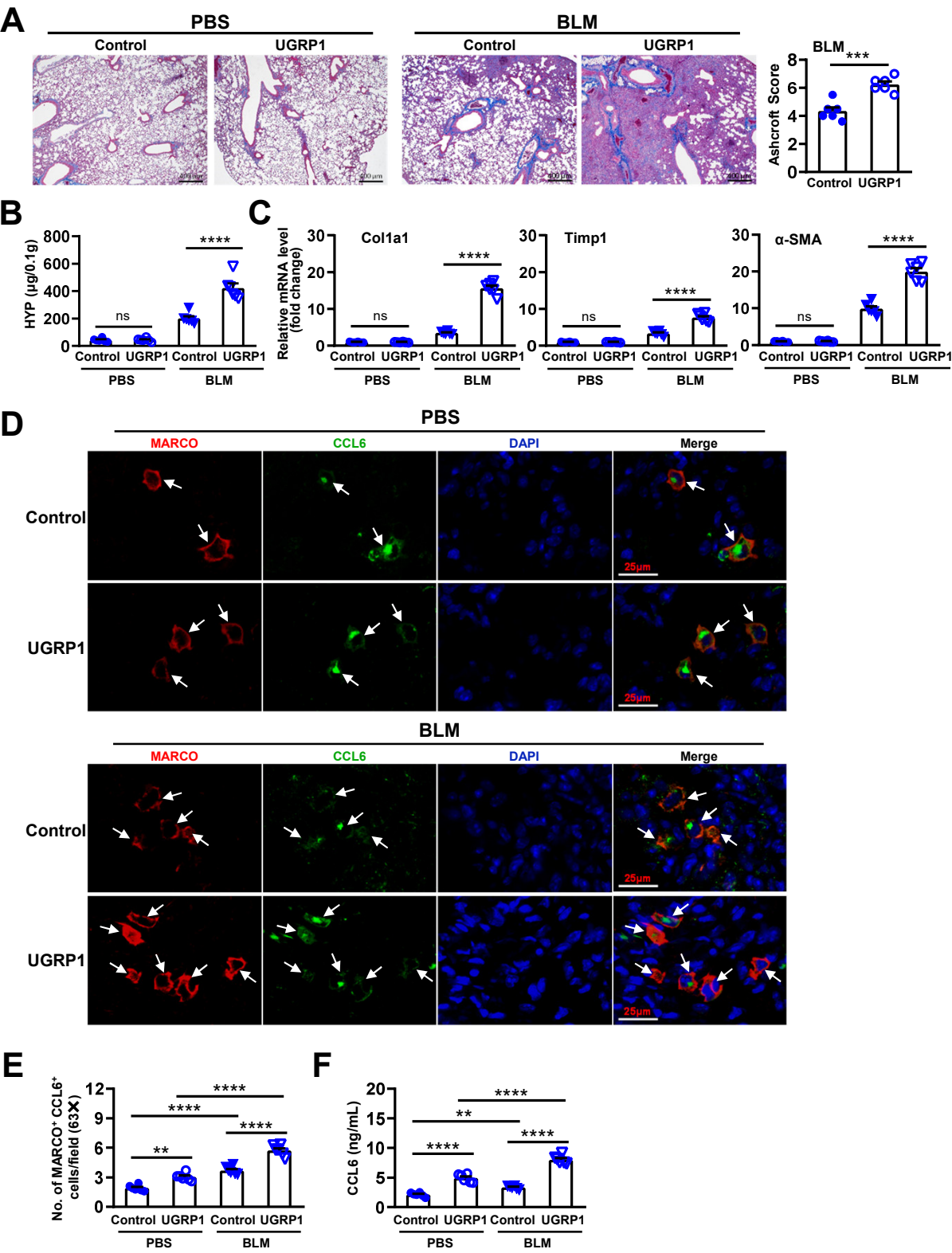

Supplement: Supplementary file 6 — Additional file 6: Figure 5. Treatment of UGRP1 protein aggravated the BLM-induced pulmonaryfibrosis of the young mice. [file 12979_2023_338_MOESM6_ESM.pdf]

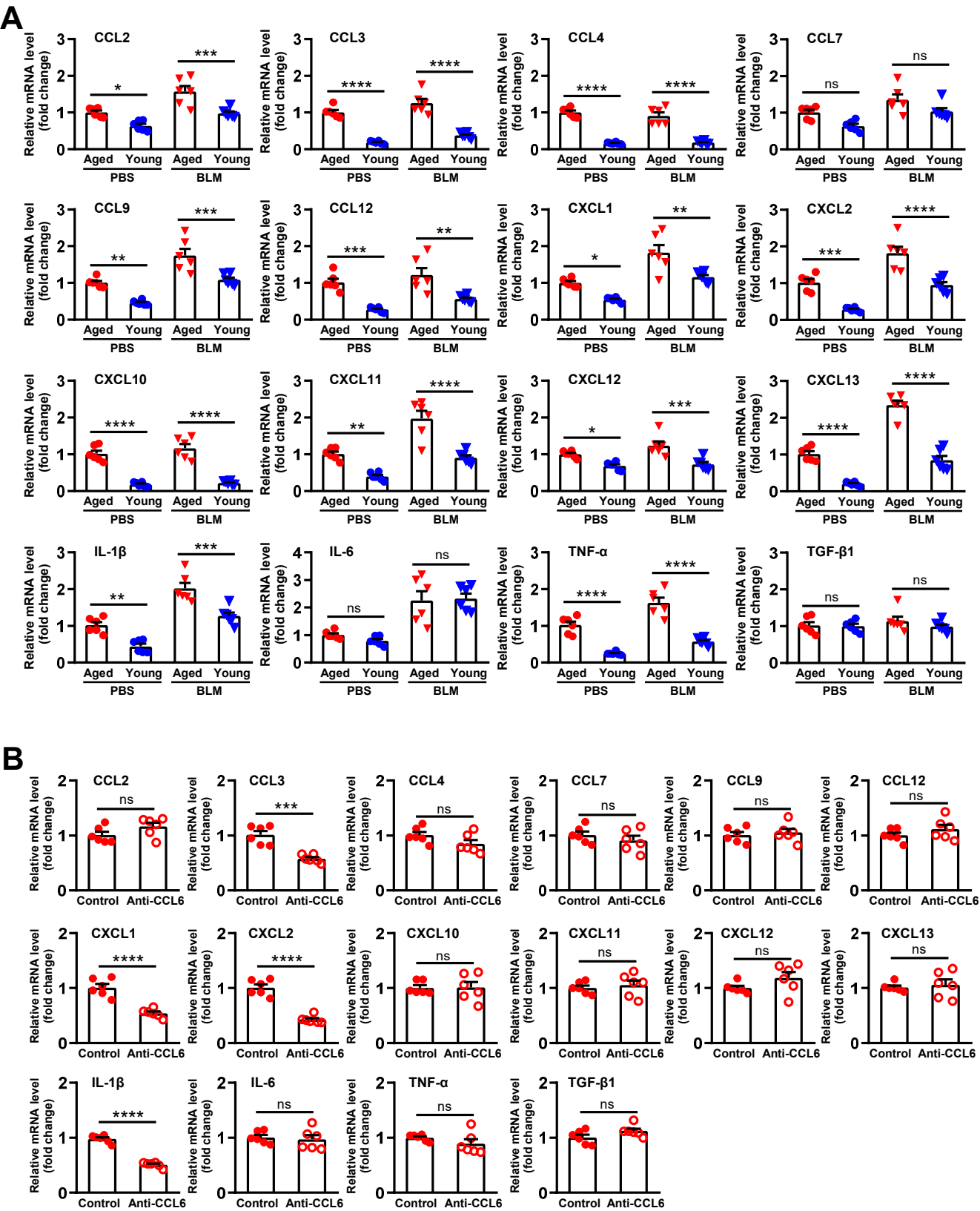

Supplement: Supplementary file 7 — Additional file 7: Figure 6. Expressions of inflammatorychemokines and cytokines in BLM-induced lung fibrosis model of aged micecompared with young mice. [file 12979_2023_338_MOESM7_ESM.pdf]
